# Super Enhanced Purification of Denatured-Refolded Ubiquitinated Proteins by ThUBD Revealed Ubiquitinome Dysfunction in Liver Fibrosis

**DOI:** 10.1016/j.mcpro.2024.100852

**Published:** 2024-10-02

**Authors:** Xinyu Cheng, Yonghong Wang, Jinfang Liu, Ying Wu, Zhenpeng Zhang, Hui Liu, Lantian Tian, Li Zhang, Lei Chang, Ping Xu, Lingqiang Zhang, Yanchang Li

**Affiliations:** 1 State Key Laboratory of Medical Proteomics, Beijing Proteome Research Center, National Center for Protein Sciences Beijing, Research Unit of Proteomics & Research and Development of New Drug of Chinese Academy of Medical Sciences, Institute of Lifeomics, Beijing, PR China; 2 School of Basic Medical, Anhui Medical University, Heifei, Anhui, PR China; 3 Department of Hepatobiliary and Pancreatic Surgery, The Affiliated Hospital of Qingdao University, Qingdao, Shandong, PR China; 4 College of Chemistry and Materials Science, Hebei University, Baoding, Hebei, PR China; 5 TaiKang Medical School (School of Basic Medical Sciences), Key Laboratory of Combinatorial Biosynthesis and Drug Discovery of Ministry of Education, School of Pharmaceutical Sciences, Wuhan University, Wuhan, PR China

**Keywords:** ubiquitinome, ThUBD, ubiquitin chains, proteomics, denatured-refolded, DRUSP

## Abstract

Ubiquitination is crucial for maintaining protein homeostasis and plays a vital role in diverse biological processes. Ubiquitinome profiling and quantification are of great scientific significance. Artificial ubiquitin-binding domains (UBDs) have been widely employed to capture ubiquitinated proteins. The success of this enrichment relies on recognizing native spatial structures of ubiquitin and ubiquitin chains by UBDs under native conditions. However, the use of native lysis conditions presents significant challenges, including insufficient protein extraction, heightened activity of deubiquitinating enzymes and proteasomes in removing the ubiquitin signal, and purification of a substantial number of contaminant proteins, all of which undermine the robustness and reproducibility of ubiquitinomics. In this study, we introduced a novel approach that combines denatured-refolded ubiquitinated sample preparation (DRUSP) with a tandem hybrid UBD for ubiquitinomic analysis. The samples were effectively extracted using strongly denatured buffers and subsequently refolded using filters. DRUSP yielded a significantly stronger ubiquitin signal, nearly three times greater than that of the Control method. Then, eight types of ubiquitin chains were quickly and accurately restored; therefore, they were recognized and enriched by tandem hybrid UBD with high efficiency and no biases. Compared with the Control method, DRUSP showed extremely high efficiency in enriching ubiquitinated proteins, improving overall ubiquitin signal enrichment by approximately 10-fold. Moreover, when combined with ubiquitin chain-specific UBDs, DRUSP had also been proven to be a versatile approach. This new method significantly enhanced the stability and reproducibility of ubiquitinomics research. Finally, DRUSP was successfully applied to deep ubiquitinome profiling of early mouse liver fibrosis with increased accuracy, revealing novel insights for liver fibrosis research.

## Highlights

• Denatured-Refolded Ubiquitinated Sample Preparation (DRUSP) combined with ThUBD is established for ubiquitinomics research.
• DRUSP can extract more ubiquitinated proteins and increase the positive identifications.
• DRUSP demonstrates superior versatility and performance coupled with pan-chains or chain-specific UBDs.
• DRUSP exhibits enhanced quantitative accuracy and reproducibility for ubiquitinomics.

## Graphical Abstract

**Figure:**
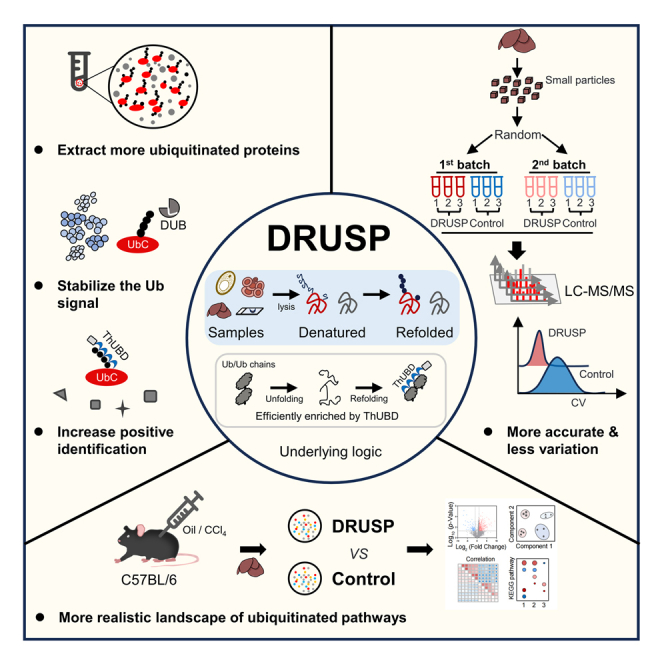

## In Brief

Ubiquitinome enrichment by artificial ubiquitin-binding domains (UBDs) hinge on the precise spatial structure of ubiquitin and ubiquitin chains with UBDs. It necessitates a non-denatured environment to sample preparation and enrichment process, which led to insufficient protein extraction, unstable ubiquitin signal, as well as abundant contaminant proteins, all of which undermine the robustness and reproducibility. This study introduces a novel methodology, DRUSP combined with ThUBD to markedly improve the efficiency of extracting and enriching ubiquitinated proteins in samples with superior reproducibility, robustness, and applicability.

Ubiquitination, a crucial posttranslational modification (PTM) in eukaryotic cells, plays a significant role in a wide range of biological processes, including protein quality control, cell cycle regulation, immune response, and stress response (1, 2). Ubiquitin exhibits a high degree of sequence and structural conservation among various species (3). The C-terminal glycine residue of ubiquitin can form covalent bonds with lysine residues in substrate proteins through a cascade of enzymatic reactions involving the ubiquitin-activating (E1), ubiquitin-conjugating (E2), and ubiquitin ligase (E3) enzymes (4). Modifying seven lysine residues (K6, K11, K27, K29, K33, K48, and K63), along with the N-terminal methionine residue (M1), carried by ubiquitin can generate eight distinct topological ubiquitin chains (5). The ubiquitin-proteasome system governs the synthesis, recognition, removal, and transmission of ubiquitin signals. Dysfunction or mutations affecting ubiquitination and related signaling pathways can contribute to various severe diseases, including cancer, immune disorders, and neurodegenerative diseases (6, 7, 8).

The profiling of the ubiquitinome facilitates a deeper understanding of the roles and functions of ubiquitination in diverse biological processes (9, 10). However, ubiquitinomics research faces several challenges. Ubiquitinated proteins constitute a relatively small proportion of the overall proteome, accounting for less than 1% of the total proteome (7). Furthermore, ubiquitinome complexity arises from mono-, multimono-, and poly-ubiquitination modifications occurring at different sites on substrates, resulting in a vast dynamic range (11). Ubiquitinated proteins with low abundance are distributed across various cellular compartments, including the extracellular matrix, membrane, cytosol, and nucleus. Additionally, the presence of highly active deubiquitinating enzymes (DUBs) and 26S proteasomes is a challenge because they can remove ubiquitination signals, rendering them unstable and variable (12, 13). To address these challenges, high-quality sample preparation for ubiquitinomics research is paramount.

The deep identification and screening of the ubiquitinome require the enrichment of ubiquitinated samples, which can be primarily classified into protein-level or peptide segment (site)-level enrichment (14). On the one hand, various enrichment materials, such as commercial TUBEs (15), tandem hybrid UBD (ThUBD) developed by our team (16), and other ubiquitin chain-specific types of UBDs (17, 18, 19, 20, 21), can be utilized for enriching ubiquitinated proteins at the protein level. Subsequently, enzymatic digestion and mass spectrometry analysis were performed to obtain ubiquitinomic data. On the other hand, high-performance immunoaffinity antibodies, such as K-ε-GG (22) or UbiSite (23), are applied for modified peptide-level enrichment. These methods possess their own advantages and complement each other for diverse scenarios.

Enrichment at the ubiquitinated protein level based on UBDs is widely used for substrate screening, detection, and crosstalk research with other PTMs. Recently, the continuous development and improvement of artificial UBDs have significantly advanced ubiquitinomics research (15, 24, 25, 26). Our team also developed and constructed a high-affinity ThUBD that can recognize eight ubiquitin chains without bias and efficiently enrich ubiquitinated proteins (16, 27). Moreover, our ThUBD has been employed for the rapid detection of ubiquitination signals by labelling with horseradish peroxidase (HRP) or fluorescein, which overcomes the detection bias of ubiquitin chains by ubiquitin antibodies (28, 29). Concurrently, ubiquitin-interacting motifs specifically enriched for K48 and K63 chains have been extensively utilized in the enrichment of specific ubiquitin chain–modified substrates in cells or tissue, allowing further exploration of the biological functions and mechanisms involved in disease occurrence and development (18, 19, 21).

However, all of these above-mentioned enrichment technologies based on UBDs encounter serious technical challenges. The recognition of ubiquitinated proteins by UBD-based materials relies on the proper spatial structure of ubiquitin and ubiquitin chains with UBDs. The hydrophobic surfaces of ubiquitin molecules, including Ile44 and Ile36, serve as the structural basis for the recognition of ubiquitin signals (3, 30). Ubiquitin chains established through distinct lysine linkages exhibit slight but varying spatial structures, thereby exposing different hydrophobic surfaces, which can be identified by specific UBDs (4). Consequently, nondenaturing conditions are required to preserve the spatial structure as much as possible, enabling UBDs to recognize ubiquitin and ubiquitin chains. However, under native conditions, the extraction of proteins from cells or tissues is insufficient, especially for insoluble samples such as fibrotic or neurodegenerative disease tissues. Additionally, highly active DUBs, the proteasome or lysosome, facilitate the removal of ubiquitination signals, leading to large variations in ubiquitinome identification and quantification. Furthermore, under native conditions, protein–protein interactions lead to the identification of numerous false-positive proteins. Given the widespread application of UBDs, overcoming these limitations and technical drawbacks is highly desirable.

In this study, we introduce a novel methodology called denatured-refolded ubiquitinated sample preparation (DRUSP), which combines ThUBD or other artificial UBDs for ubiquitinomics research. This approach offers a groundbreaking solution to overcome the aforementioned challenges and issues, substantially enhancing the efficiency of ubiquitinated protein extraction and enrichment in samples with improved stability, reproducibility, and suitability. As a result, this approach enables the efficient enrichment and precise quantification of ubiquitinated proteins in diverse samples. By applying this method, we investigated ubiquitination changes in an early liver fibrosis model, leading to the identification of upregulated ubiquitinated proteins with increased accuracy and providing a more precise global ubiquitination landscape in the early liver fibrosis model.

## Experimental Procedures

### Animals

C57BL/6 male mice aged 6 to 8 weeks were purchased from SPF Biotechnology. All mice were maintained in a pathogen-free environment with moderate humidity and temperature. This project was approved by the Institute Animal Care and Use Committee of Beijing Proteome Research Center (ethics review number: IACUC-20200303-08MO) (31). The mice were treated and euthanized in accordance with the Institutional Guidelines for Animal Care and Welfare. To minimize potential contamination of livers by blood proteins, perfusion was conducted after the mice were euthanized. Subsequently, the mouse livers were dissected, rinsed twice with cold PBS, and promptly frozen in liquid nitrogen. The samples were stored at −80 °C until protein extraction.

### Cell Culture and Harvesting

The yeast strain JMP024, in which the endogenous ubiquitin gene was knocked out and the synthetic ubiquitin gene was expressed with polyhistidine and myc tags, was used in this study (32, 33). In general, the yeast cells were cultured in YEPD media (1% yeast extract, 2% peptone, and 2% dextrose) at 30 °C with rotation at 220 rpm and harvested in the early log phase (A_600_ = 1.0). The supernatant was removed by centrifugation at 2000*g* for 4 min at 4 °C. The cell precipitates were washed with 0.1% sodium azide to better inhibit growth and stored at −80 °C until lysis.

The human embryonic renal epithelial cell line (HEK293T) was cultured in Dulbecco's modified Eagle's medium supplemented with 10% fetal bovine serum and 1% penicillin/streptomycin. After reaching a cell density of approximately 95% in an incubator with 5% CO_2_ at 37 °C, the culture medium was removed, and the cells were washed twice with cold PBS. The cell pellets were then collected by centrifugation at 300*g*.

### Protein Extraction

For the establishment and verification of the method, the mouse liver tissue was lysed using three different lysate conditions (strong, middle, and weak buffers, the components were listed in Supplemental Fig. S1*A*), with the addition of 1 ml of lysate for every 0.2 mg of tissue. The cryogenic freeze grinder (Shanghai Jing Xin) was utilized to grind the sample 10 times using steel balls, vortex for 30 s, stopped for 30 s, and repeated 10 cycles. After grinding, the steel balls were removed, and low-temperature ultrasound was performed using a noncontact ultrasonic machine (SCIENTZ) for 10 min (worked for 4 s, stopped for 4 s with a total time of 10 min at an intensity of 80%).

The yeast cells were lysed by glass beads (Sigma) in lysis buffer (strong buffer and weak buffer) and were placed in a cryogenic freeze grinder, vortexed for 30 s, stopped for 30 s, and repeated 30 cycles. The precooled lysis solution was added to the mammalian cell pellets, and after cell suspension, the cells were lysed at low temperature using a noncontact ultrasonic crusher (operated for 4 s, paused for 4 s, with a total working time of 10 min and a power of 80%).

After ultrasonic cracking, lysates were centrifuged at 21,000*g* at 4 °C for 10 min to eliminate insoluble debris, and the supernatant was utilized for the subsequent detection of ubiquitination signal strength. All these steps were conducted under low temperatures to prevent protein degradation. However, solutions containing urea and SDS should be avoided on ice for a long time to avoid precipitation of urea and SDS.

For protein extraction of FFPE samples, all samples were scraped off the slides and transferred into centrifuge tubes. After instantaneous centrifugation, 1 ml of xylene was added, followed by shaking at 300 rpm for 10 min at room temperature. The samples were then centrifuged at 12,000*g* for 3 min to remove the supernatant and then repeated three times with xylene to complete dewax. Subsequently, rehydration was carried out using an anhydrous ethanol gradient (100%, 96%, 70%, 0%). The samples were rotated and mixed for 5 min, followed by centrifugation at 12,000*g* for 3 min. The supernatant was then dried using a vacuum rotary dryer for 10 min, and then, the lysis buffer (strong and weak buffers) was added. After 20 min of noncontact ultrasound, the samples were shaken at 300 rpm at 99 °C for 1 h. Finally, the samples were centrifuged at a low temperature of 17,000*g* at 4 °C for 6 min, and the supernatant was collected to obtain the total protein lysates.

### Refolded Proteins and UbC Affinity Purification

For the DRUSP method, proteins were extracted from strong and middle lysis buffers. To remove urea, SDS, and sodium deoxycholate from the solution, which affects subsequent affinity purification, a 10 kDa ultrafiltration tube (Millipore) was used to carry out gradient displacement to achieve refolding. In simple terms, solutions containing different concentrations of urea and no detergent were successively added (buffer 1 with 8 M urea, buffer 2 with 4 M urea, buffer 3 with 2 M urea, buffer 4 without urea; all buffers contain 50 mM Tris–HCl, pH 7.5, 150 mM NaCl, 0.1% NP-40, 10% glycerol, 2 mM DTT) and then placed in a 4 °C centrifuge for centrifugal ultrafiltration. Buffer 4 was continually added to the centrifuged liquid until the urea concentrations remained lower than 0.1 M. Subsequently, the refolded proteins were incubated with ThUBD affinity medium in buffer 4 solution for 2 h at 4 °C. Then, the purified ubiquitinated conjugates were eluted with 2× SDS loading buffer and detected by SDS-PAGE and Western blotting.

For the traditional method (Control), proteins were extracted with weak buffer, incubated with ThUBD affinity medium for 2 h at 4 °C, and then eluted. Before Western blotting and LC-MS/MS analysis, 10 mM TCEP and 40 mM CAM were added for reduction and alkylation at room temperature for 15 min. In specific UBD enrichment experiments, M1-TUBE, K48-UIM, and K63-TUBE were used under the DRUSP and Control methods.

### SDS-PAGE and Western Blot Analysis

Protein concentration of the supernatant was determined using a bicinchoninic acid (BCA) protein assay (Thermo Fisher Scientific). Lysates were prepared for separation by adding SDS loading buffer [250 mM Tris–HCl (pH 6.8), 20% (v/v) glycerol, 0.5% (w/v) SDS, 0.02% (w/v) bromophenol blue], followed by resolution *via* SDS-PAGE. Subsequently, the protein samples were electrophoretically transferred onto a nitrocellulose membrane using the Bio-Rad Trans-Blot SD Semi-Dry Transfer System at 15 V for a duration of 15 to 55 min. Ponceau S staining or silver staining served as the loading control. Membranes were blocked with a solution of 10% nonfat milk in TBST [20 mM Tris–HCl (pH 7.4), 150 mM NaCl, 0.02% Tween-20] for 1 hour. Following blocking, the membranes were incubated with ThUBD-HRP (1 μg/μl) diluted to a ratio of 1:1000 at room temperature for 2 hours. Excess ThUBD probe was removed through three 10-min washes with TBST. The membranes were then exposed to Super Signal West Pico chemiluminescent substrate (Thermo Fisher Scientific) for 1 min at room temperature and visualized using a Tanon 5200 chemiluminescence imaging system (Tanon). Band intensities were detected and quantified using ImageJ 1.48v software (National Institutes of Health).

For the specific detection of M1-, K63-, and K48-linked polyubiquitinated proteins, the following antibodies were utilized: anti-linear chain (M1-specific) antibody (Lifesensors, #AB130, clone LUB9), anti-Ub-K48 antibody (Millipore, #05-1307, clone apu2), and anti-Ub-K63 antibody (Millipore, #05-1308, clone apu3). Unlike ThUBD-HRP, the use of these antibodies necessitates an additional incubation step with the corresponding secondary antibody.

### LC‒MS/MS Detection and Data Analysis

All samples were digested with trypsin overnight and desalted using an in-house packed C_18_ StageTip before LC-MS/MS analysis. After LC-MS/MS detection, all the raw files were searched by MaxQuant (version 2.1.4.0) against the Swiss-Prot reviewed mouse database (released on 2024_02_03, containing 17,201 entry proteins). Then, the data were analyzed in Perseus (version 2.0.3.0). All the data were statistically tested, and *p* < 0.05 was considered to indicate a significant difference. More detailed information can be found in the Supplement Material.

### Ubiquitin and Ubiquitin Chains Analysis

The eight types of di-ubiquitin chains (Lifesensors) were used for analysis. Di-ubiquitin chains were separated through 13.5% SDS-PAGE and then subjected to silver staining and western blotting. For each test, 20 ng of ubiquitin chains were used. The di-Ub chains were digested by trypsin and subjected to LC-MS/MS analysis.

For selective reaction monitoring (SRM) detection, the resulting peptide samples were dissolved in a mass spectrometry (MS) sample resuspending buffer (1% ACN/1% FA) and analyzed by LC-MS/MS platform as described before (16, 34, 35). Peptides were detected by the Orbitrap mass spectrometer in a survey scan (300–1600 m/z, resolution 30,000) followed by SRM scans in the LTQ for eight ubiquitin linkages. The specific parameters and peptide information of SRM can be found in supplementary Table S1. The intensities of peptides were manually analyzed by ion chromatograms using Xcalibur v2.0 software (Thermo Finnigan).

### Experimental Design and Statistical Rationale

The aim of this study is to establish a new method for highly efficient enrichment of ubiquitinated proteins. We conducted a comprehensive evaluation and comparison of all aspects of the method, detailing the experimental procedures in each respective section (Supplemental Fig. S1). All evaluations of the methods were compared with traditional Control methods. When measuring protein concentrations using BCA, three techniques were repeated for each condition to calculate statistical differences. For the comparison of the DRUSP and the control method omics identification, there are two technical duplications. Between technique repeats were independent experiments, and the steps from protein extraction to mass spectrometry detection are performed independently. The significance of the difference in signal intensity between the proteins and K-ε-GG peptides identified by the two methods was tested using a nonparametric Mann-Whitney test. The statistical differences in Ub FOT were examined using a double-tailed student *t* test between the two techniques. For the establishment of CCl_4_-induced liver fibrosis mouse model and subsequent experiments, three biological replicates were used for CCl_4_ group and Oil group, respectively. The statistical differences were examined using a double-tailed student *t* test. For the ubiquitinome of CCl_4_-induced mouse liver fibrosis, to ensure strict analysis, proteins quantified in more than 30% of samples of two groups were used for subsequent bioinformation analysis, and the analysis of differential proteins selected only 12 quantitative proteins as conditions for screening. For Western blotting analysis, the batch experiments were calculated from six replicates, and model validation was generated from three biological replicates. For SRM analysis of ub chains, the intensities of peptides were analyzed by ion chromatograms using Xcalibur v2.0 software (Thermo Finnigan) and two technical repeats were applied. *p*-value less than 0.05 were significant and indicated by asterisks as follows: ∗ *p*-value <0.05, ∗∗ *p*-value <0.01, ∗∗∗ *p*-value <0.001, ∗∗∗∗ *p*-value <0.0001; NS, not significant.

## Results

### The DRUSP Method can be Used to Extract More Ubiquitinated Proteins

**Fig. 1:**
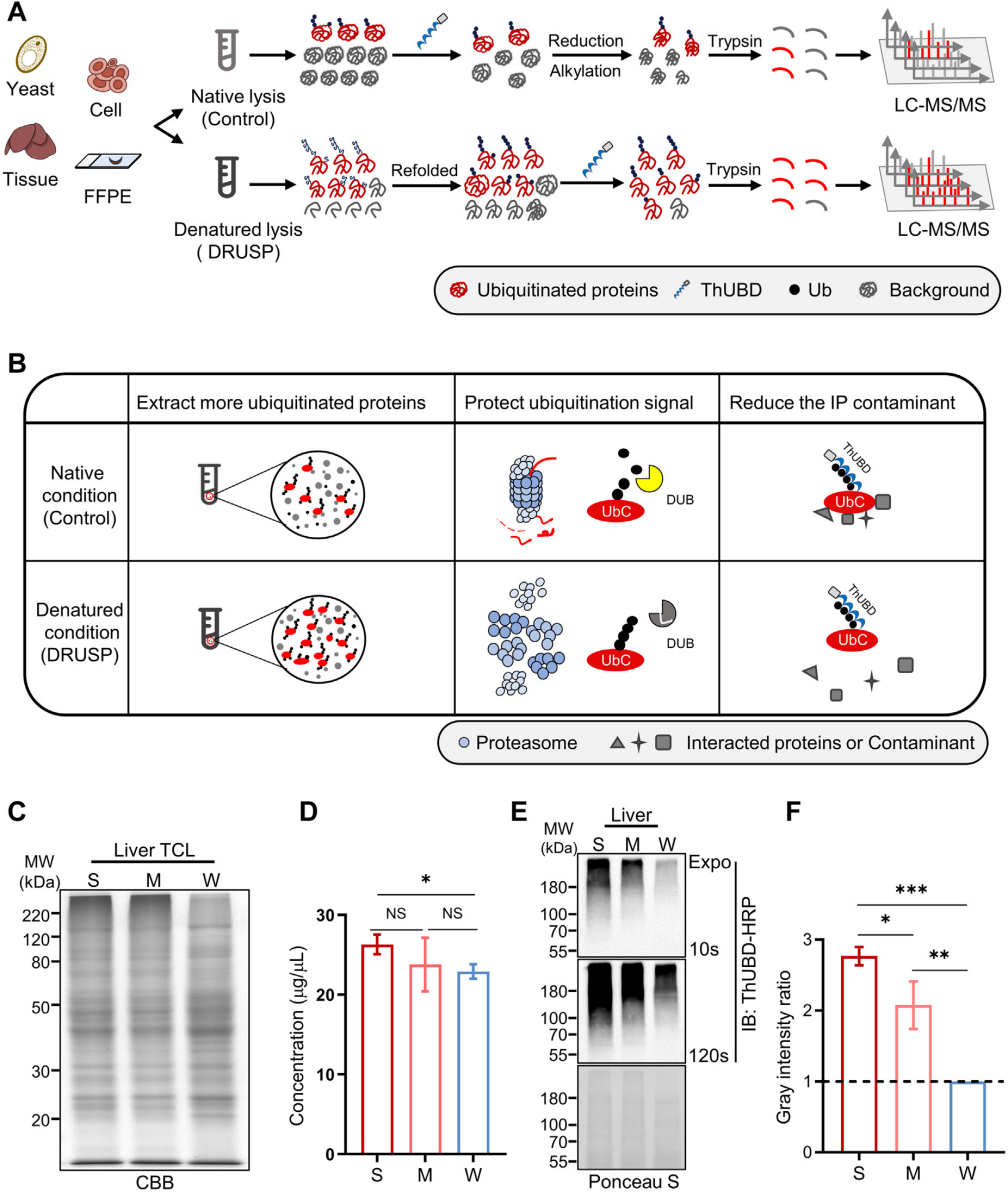
**Schematic representation of the experimental design and more ubiquitination signals could be obtained under denatured condition**. *A*, comparison of the Control (traditional) and newly developed DRUSP approaches for ubiquitinomics research. *B*, comparison of the advantages of Control and DRUSP methods. DRUSP could extract more proteins (*left*), protect the ubiquitin signal from proteasomes or deubiquitinases (*middle*), reduce contaminants, and increase the percentage of positive UbCs (*right*) in the enriched samples. *C*, the denatured method could extract slightly more proteins. The quality of protein extracted from mouse liver was determined by Coomassie bright blue (CBB) staining under three conditions: strong (S), middle or moderate (M), and weak (W). Equal amounts of liver samples were subjected to lysis buffer and prepared in parallel. The strong buffer contained 8 M urea and different detergents, the middle contained 8 M urea but with fewer detergents, and the weak buffer contained native condition without denatured agents but with glycerol. The detailed components were listed in Supplemental Fig. S1*A*. *D*, protein concentrations of mouse liver samples extracted using S, M, and W buffers were estimated using a BCA assay. The graphs show the means ± S.D. (n = 3), ∗ *p*-value <0.05. *E*, immunoblot analysis of ubiquitin signals under different extraction conditions. Ponceau S was used as a loading control and blotted with ThUBD-HRP probe to detect ubiquitin signals. *F*, quantification of the ubiquitin signals in image panel *E*. The data represent the average of three independent experiments and were analyzed using a two-tailed paired *t* test. The graphs show the means ± S.D. (n = 3) ∗*p*-value <0.05, ∗∗ < 0.01, ∗∗∗ < 0.001. NS, not significant.

In the field of ubiquitinomics research, purifying ubiquitinated proteins from biological samples at the protein level often involves the use of artificially engineered materials containing ubiquitin binding domains (UBDs). This enrichment process includes extracting proteins under native conditions, co-incubating them with UBDs to capture ubiquitinated proteins, and subsequently performing reduction, alkylation, and enzymatic digestion for detection *via* mass spectrometry (MS) (Fig. 1*A*, upper). However, the classical method of purifying ubiquitinated proteins under native conditions is limited by challenges such as insufficient protein extraction, degradation of ubiquitin signals, and abundant contaminants (Fig. 1*B*, up). To overcome these drawbacks, we developed Denatured-Refolded Ubiquitinated Sample Preparation (DRUSP) combined with ThUBD for ubiquitinated conjugate (UbC) purification (Fig. 1*A*, bottom). Under strongly denatured conditions, the proteins impeded in the samples could be extracted thoroughly. Theoretically, the activity of DUBs, proteasomes or other proteinases could be effectively inhibited, and protein-protein interactions could be disrupted (Fig. 1*B*, bottom). To restore the spatial structure of ubiquitin and its chains, we employed a method of low-temperature ultrafiltration buffer exchange to facilitate the efficient and gradual refolding of ubiquitinated proteins. After ThUBD enrichment, the ubiquitinated proteins were digested before LC-MS/MS analysis.

Initially, we selected three types of lysis buffers with varying degrees of denaturation in terms of highly (Strong, S), moderately denatured (Middle, M) and native conditions (Weak, W). The components of the three buffers were shown in the diagram, with or without the addition of components such as urea, SDS or sodium deoxycholate detergents (Supplemental Fig. S1*A*). In strong (S) and middle (M) denaturing lysis buffers, TCEP and CAM were included to facilitate immediate reduction and alkylation of proteins to inhibit enzyme activity, thereby protecting ubiquitin signals. Equivalent amounts of fresh mouse liver tissue were chosen to test the experimental workflow (Fig. 1*A*). After parallel protein extraction under the three conditions, an equal volume of sample was separated by 10% SDS-PAGE. The concentration and purity of the proteins were determined using Coomassie brilliant blue staining (Fig. 1*C*). Although there was general consistency among the protein bands under the three conditions, a significantly greater abundance of high-molecular-weight regional proteins was observed under denatured conditions (Fig. 1*C*). The BCA assay also revealed that the protein concentration extracted with strong buffer was significantly greater than that extracted with weak buffer (Fig. 1*D*).

Immunoblotting analysis demonstrated that the ubiquitin signals were most abundant in the strong buffer extraction group, followed by those in the moderate buffer extraction group and those in the weak buffer extraction group (Fig. 1*E*). This increase was nearly 3-fold greater under denatured conditions than under native conditions (Fig. 1*F*). The three types of samples were subsequently refolded and detected (Supplemental Fig. S1, *B* and *C*). Minimal protein loss occurred under strongly denatured and natural conditions, while moderate conditions led to more substantial precipitation (Supplemental Fig. S1*C*). Western blotting revealed no significant change in ubiquitin signals before and after renaturation under strong denaturing buffer; however, varying degrees of loss were observed under moderate and weak denaturing buffer conditions (Supplemental Fig. S1*D*). Therefore, we selected strongly denatured conditions as the optimal conditions for subsequent experiments, and parallel comparisons with native conditions were performed. These two conditions were also used for yeast cells, mammalian 293T cells, and FFPE tissues. There were more ubiquitin signals under strong conditions for any type of sample (Supplemental Fig. S2, *A*–*C*).

### The DRUSP Method Successfully Restored Eight Ubiquitin Chains, Which Were Efficiently Recognized and Enriched by ThUBD

**Fig. 2:**
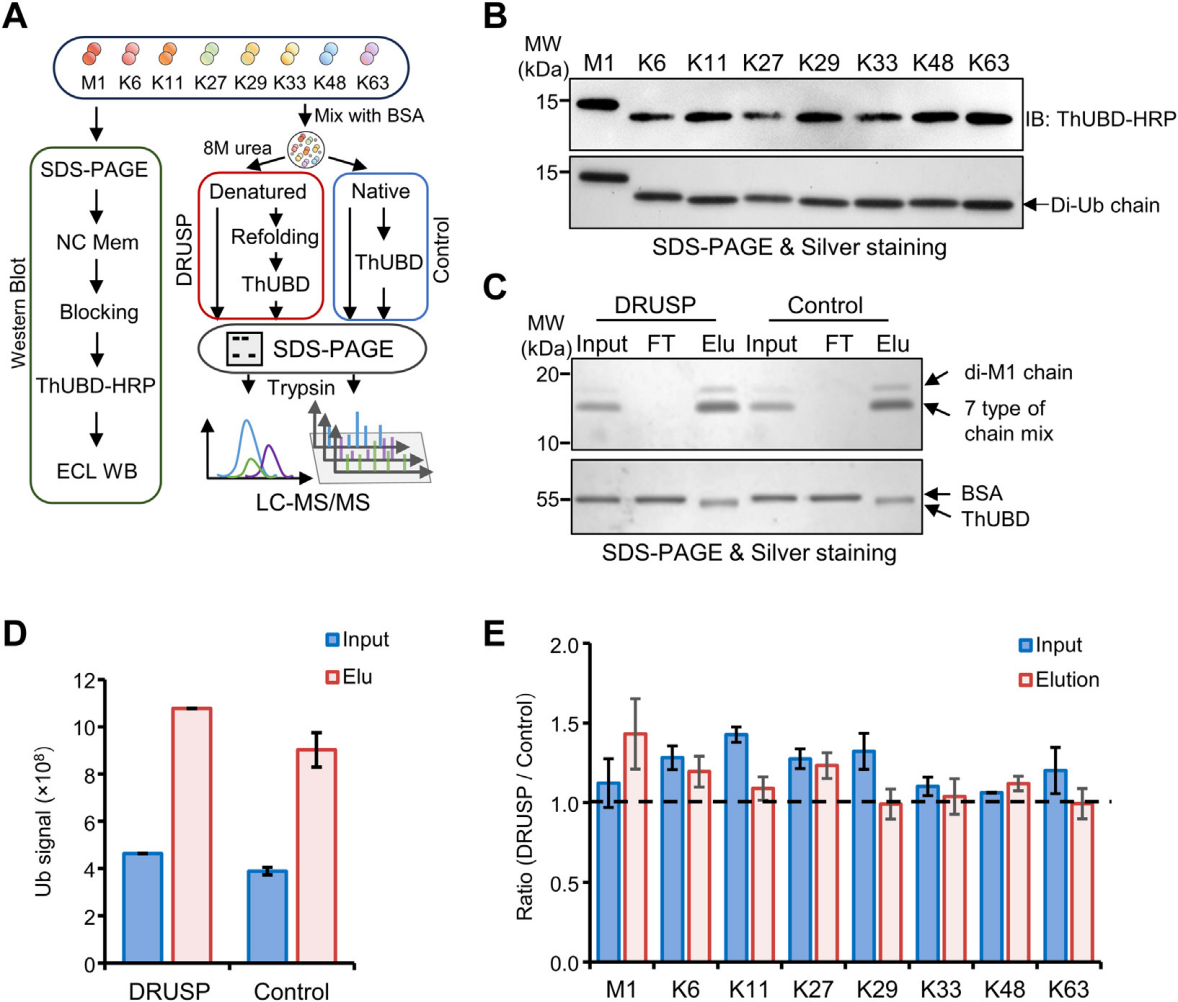
**All eight ubiquitin chains could be restored under DRUSP method and efficiently enriched by ThUBD**. *A*, workflow for verifying the refolding capacity and enrichment efficiency of eight ubiquitin chains after DRUSP method. These results were confirmed and detected by WB and affinity experiments under DRUSP and Control methods. The eight ubiquitin chains were mixed and divided into two equal parts. Affinity purification was performed by using ThUBD, one part by DRUSP method and the other under Control conditions. *B*, immunoblot analysis demonstrated the ability of ThUBD to identify eight ubiquitin chains. Equal amounts of commercial di-Ub chains were separated by SDS‒PAGE, and silver staining was used as a loading control. *C*, the di-ubiquitin chain mixture (Input), flow-through (FT), and elution (Elu) were detected by silver staining. Unbound BSA and eluted ThUBD were used as controls. Although the molecular weights of BSA and ThUBD are similar, they are still distinguishable. *D*, quantification of the ubiquitin signal before and after enrichment in the DRUSP and Control groups. The total ubiquitin signal was detected by MS SRM method through detecting the signal of the S57 peptide (TLSDYNIQK) of ubiquitin produced by trypsin digestion. Two technical replicates were performed. *E*, the ratio (DRUSP/Control) of eight ubiquitin chains before and after enrichment. The resulting peptides were detected by MS SRM method. The graphs show the means ± S.D. (n = 2) derived from two technical replicates.

Ubiquitin is considered a small molecule protein that exhibits excellent thermal stability (36). Furthermore, ubiquitin also possesses the distinct characteristics of a highly structured natural state and remarkable self-folding ability (37, 38). Due to the absence of cysteine and disulfide bonds, its folding rate is fast (39, 40). Nevertheless, whether these characteristics can be extended to eight ubiquitin chains remains unknown. To ascertain whether ubiquitin chains can also regain their appropriate spatial structures and be recognized by ThUBD, we conducted a validation experiment using a simplified system following the workflow (Fig. 2*A*). First, eight commercial di-ubiquitin chains were separated by SDS-PAGE and blotted with a ThUBD-HRP probe. The results demonstrated that the ThUBD probe could accurately identify eight ubiquitin chains (Fig. 2*B*). Subsequently, we mixed eight chains with BSA and divided them into two equal portions. One portion was denatured with 8 M urea and refolded as described previously, while the other portion was left untreated. Both samples were then enriched and purified using equal amounts of ThUBD beads. The effectiveness of purification was assessed by silver staining. The bands at the same position of the di-ubiquitin chains were both input and eluted, while only the unbound BSA band was present in the FT, indicating successful enrichment by ThUBD of only eight ubiquitin chains (Fig. 2*C*). MS SRM confirmed consistent overall ubiquitin signals and that each ubiquitin chain was present at a ratio of approximately 1 before and after enrichment under the two conditions (Fig. 2, *D* and *E* and Supplemental Table S1). These findings preliminarily demonstrated that the DRUSP method could restore the appropriate spatial structure of eight denatured ubiquitin chains and that the chains could be recognized and enriched by ThUBD with efficiency comparable to that of the native state.

To further confirm whether this restoration of appropriate spatial structure occurs in complex systems, we conducted *in vitro* deubiquitinating reaction experiments using the nonspecific DUB USP21 in mouse liver whole-cell lysates. The immunoblotting results showed that USP21 could cleave ubiquitinated proteins prepared by DRUSP or traditional Control methods, thus demonstrating the ability of the ubiquitin chains to restore a suitable spatial structure for recognition by DUB (Supplemental Fig. S3*C*).

To confirm the stability of the ubiquitin signals after refolding, equal amounts of mouse liver proteins were prepared by DRUSP and control methods and subsequently incubated at 37 °C for 0, 0.5, 1, 2, or 4 h. The results showed that there was nearly no significant change in the ubiquitin signals within 4 h compared to the initial time point (0 h) in the DRUSP samples. However, under native conditions, the ubiquitin signals gradually weakened and nearly disappeared within 4 h when common inhibitors were added. These results proved that the DRUSP method could significantly hinder the activity of enzymes and increase the stability of ubiquitin signals (Supplemental Fig. S3, *A* and *B*).

### DRUSP Combined with ThUBD for UbC Enrichment Exhibited Superior Enrichment Efficiency and Broader Applicability

**Fig. 3:**
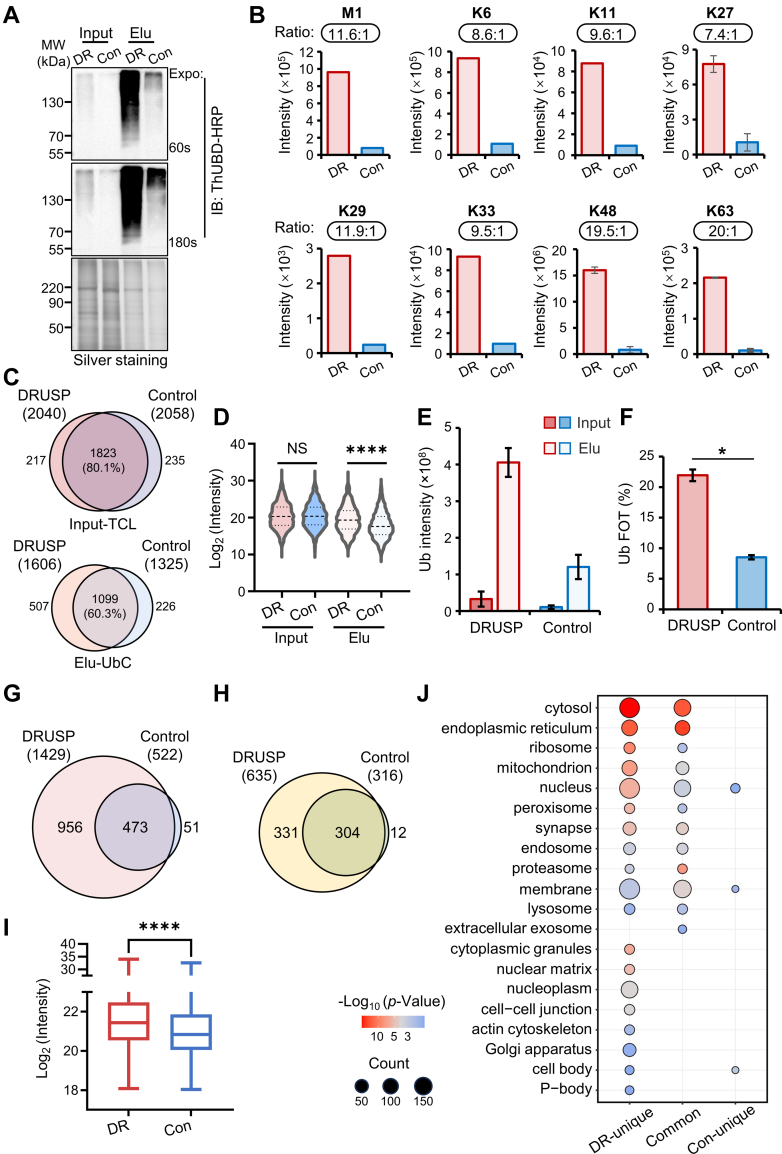
**The DRUSP method could purify more UbC than the traditional Control method**. *A*, immunoblot analysis of the ubiquitin signal derived from DRUSP and Control methods. Equal amount of starting mouse liver was used for these two methods. Silver staining was used as a loading control. *B*, the intensity ratios (DRUSP/Control) of eight ubiquitin chains in elution samples derived from DRUSP and Control methods were determined by LC-MS/MS. *C*, overlaps of the proteins identified from the whole proteome (TCL, *top*) and ubiquitinome (UbC, *bottom*) based on the DRUSP and Control methods. *D*, violin diagram of the log_2_ protein intensity distribution for each group. Input represents the whole proteome, and Elu represents the ubiquitinome group. ∗∗∗∗*p*-value <0.0001, NS, not significant. *E*, the absolute signal of ubiquitin for each group. The graphs show the means ± S.D. (n = 3) derived from three independent experiments. *F*, the total fraction (FOT) represents the intensity proportion of ubiquitin to all proteins in the enriched sample. ∗*p*-value <0.05. *G*, Venn diagram illustrates the intersection of identified diGly peptides between the DRUSP and Control approaches. The tryptic elution samples derived from DRUSP and Control methods were subjected to K-ε-GG antibody enrichment, respectively. *H*, Venn diagram of proteins corresponding to diGly sites according to the DRUSP and Control methods. *I*, the box plot shows the log_2_ intensity distribution of diGly peptides identified by the DRUSP and Control methods. ∗∗∗*p*-value <0.001. *J*, dot plot shows the CC of Gene Ontology terms enriched in (*I*). The size of the dot is based on the number of genes enriched, and the color of the dot shows the enrichment significance.

To further investigate the efficiency of the DRUSP method on complex samples, we employed ThUBD to enrich the UbC of mouse liver samples. Similarly, we compared the DRUSP to Control methods. Compared to the total proteins (Input), the elution samples exhibited noticeable ubiquitination signal enrichment, while the FT was considerably reduced (Supplemental Fig. S4*A*). The enrichment of ubiquitin signals under DRUSP method was significantly greater (Fig. 3*A*). LC-MS/MS revealed a significantly greater enrichment of eight ubiquitin chains under DRUSP method, with an approximately 10-fold increase compared to that of Control method (Fig. 3*B*).

LC-MS/MS analysis was performed on the proteome and enriched ubiquitinome samples. The results revealed minimal differences in the identified amounts of proteins between the two methods, which identified 2040 protein groups in DRUSP and 2058 in the control method. Among these, 1823 proteins overlapped, accounting for 80.1% (Fig. 3*C*, top). The ubiquitinome group identified 1606 and 1325 potential ubiquitinated proteins, and only 1099 overlapped, accounting for 60.3% (Fig. 3*C*, bottom). There was a consistent distribution observed in the whole proteome with the same log_2_ value of median signal intensity under the two conditions, indicating no significant difference at the whole protein level (Fig. 3*D*). However, there were significant differences in the ubiquitinome between DRUSP group (2.8-fold greater) and Control group (*p* < 0.001) (Fig. 3*D*). Correlation analysis of the overall data distribution revealed that the proteome correlation coefficient R^2^ reached 0.8, surpassing that of the ubiquitinome group (R^2^ = 0.5). Additionally, the log_2_ ratio distribution of the proteome more closely followed a normal distribution, with a mean value close to 0. Conversely, the distribution within the ubiquitinome group exhibited a significant rightward shift, with an average value exceeding 1.5. The SD for the proteome was 1.2, while for the ubiquitinome, it exceeded 2.1. These results indicated a greater degree of dispersion and substantial differences within the ubiquitinome than minimal variations across the proteome (Supplemental Fig. S4, *B* and *C*). Furthermore, despite few disparities in overall identified amounts, under denatured conditions, a greater absolute ubiquitin signal intensity was observed at both the proteome and ubiquitinome levels (Fig. 3*E*). There was an approximately one-fold increase in the proteome and a greater than 3-fold increase in the ubiquitinome. Moreover, under denaturing conditions, there was an approximately 2.4-fold increase in the overall signal proportion (FOT) in the ubiquitinome (Fig. 3*F*), indicating that a high percentage of UbC was derived from DRUSP.

The significant difference observed in Figure 3*A*, where the DRUSP method showed a markedly higher ubiquitination signal than the Control method, contrasts with the similar number of proteins identified in Figure 3*C*. To further explain this discrepancy, we conducted an additional analysis of the data. Upon examining the individual fractions of both the DRUSP and Control groups, we observed no significant disparity in the number of protein identification, a pattern consistent across both input and elution samples (Supplemental Fig. S5, *A*–*E*). Notably, the signal intensity for ubiquitin and ubiquitin chains (*e.g.* K48, K11, K63) in the elution fraction exhibited a marked disparity, with the DRUSP method demonstrating a signal strength of one order of magnitude greater or more than the control, particularly in the F1 and F2 fractions, which correspond to the high MW ubiquitin smear on WB (Supplemental Fig. S5, *F*–*I*). Among the limited number of GG-sites identified, the DRUSP method identified a greater number of sites with enhanced signal intensity than the control conditions (Supplemental Fig. S5, *J*–*L*). Given the paucity of identified sites, we further enriched diGly-modified peptides from the corresponding samples and identified 1429 and 522 ubiquitinated sites, corresponding to 635 and 316 ubiquitinated proteins, respectively (Fig. 3, *G* and *H* and Supplemental Table S2). The DRUSP method was found to enrich ubiquitin peptides to 1.73-fold higher than the Control, accompanied by a stronger overall signal at the ubiquitinated sites. The majority of the proteins and sites enriched under Control method overlapped with those enriched by DRUSP. The median overall sites intensity was approximately one-fold greater under DRUSP method than under Control, with a significant difference between the two groups (*p* < 0.001) (Fig. 3*I*). Furthermore, Gene Ontology analysis of Cellular Components was conducted on unique and common ubiquitinated proteins from panel I. Most of these proteins were associated with various cellular components, such as the cytosol, membrane, endoplasmic reticulum, mitochondria, and nucleus. However, there were more and more types of proteins present under the DRUSP method, including proteins that were difficult to extract, such as those in the actin skeleton and neuron cell body (Fig. 3*J*). In summary, the DRUSP approach not only improved enrichment efficiency, but also provided more comprehensive ubiquitinomic information.

### The DRUSP Method is Also Applicable for Sub-ubiquitinome Purification by Ubiquitin Chain-Specific UBDs

**Fig. 4:**
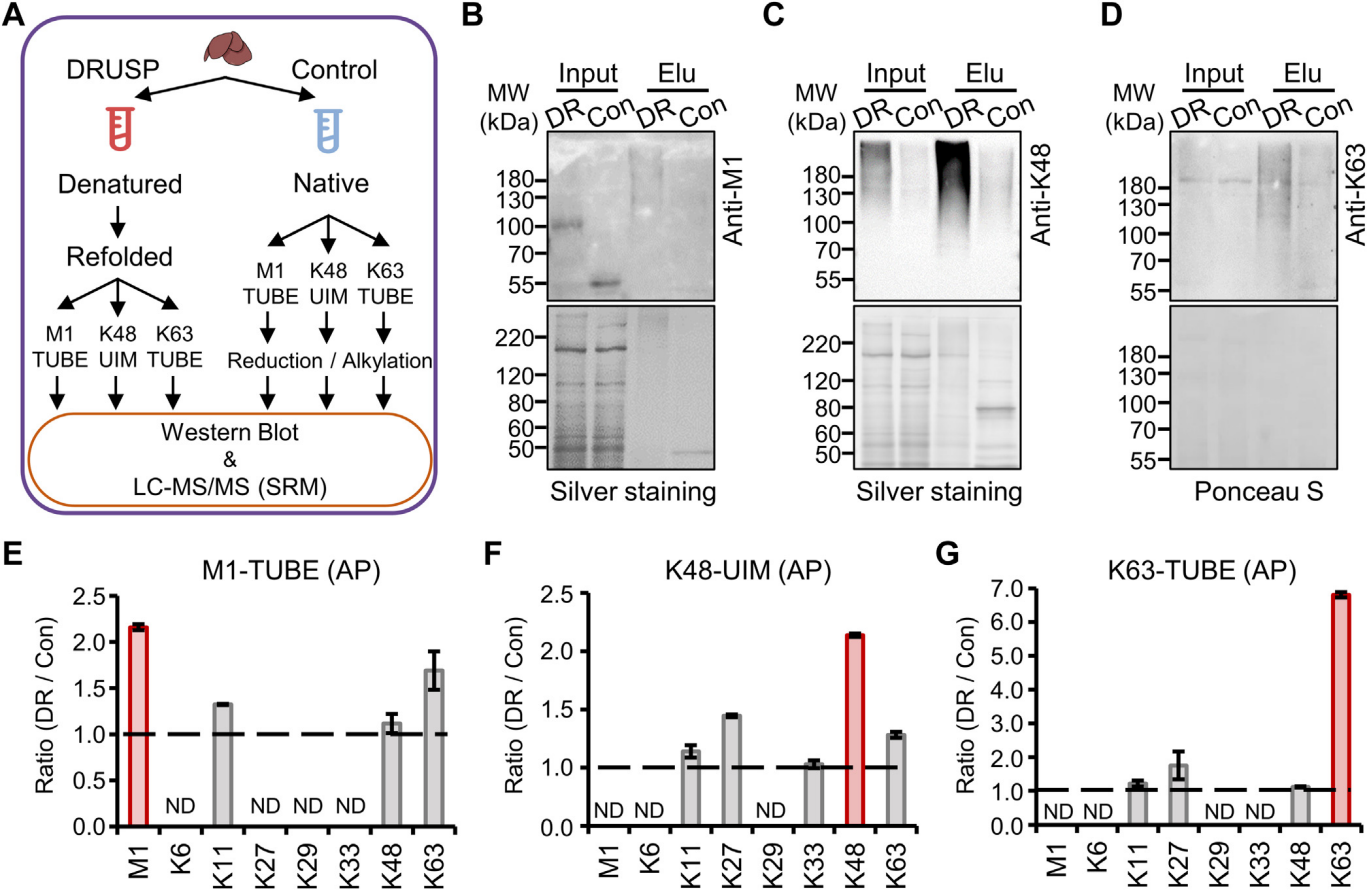
**DRUSP combined with ubiquitin chain-specific UBDs could promote sub-ubiquitinome enrichment and profiling**. *A*, workflow of different linkage-specific UBDs, such as M1-TUBE, K48-UIM, and K63-TUBE, used to enrich chain-specific modified substrates under DRUSP and Control methods. All eluents were verified by WB analysis and MS SRM quantification. *B*–*D*, immunoblot analysis of ubiquitin signals before and after enrichment with M1-TUBE, K48-UIM, and K63-TUBE based on the DRUSP and Control methods, respectively. *E*–*G*, the intensity ratio (S/W) of the eight ubiquitin chains enriched with different UBDs, with *red* representing specific chains and *gray* representing other chains. The signal intensities of eight ubiquitin chains were generated by the peak area of the spectrum detected by MS SRM method.

Using the DRUSP method, we demonstrated that eight ubiquitin chains can recover their appropriate spatial structures and can be recognized and enriched by ThUBD. The workflow of the DRUSP method effectively protected all ubiquitin chains from degradation or removal, ensuring the most authentic state of ubiquitin signals in biological samples. Therefore, the combination of the DRUSP method with ubiquitin chain-specific UBDs can be utilized for the enrichment and detection of specific ubiquitin chain-modified substrates. To validate the feasibility and universality of DRUSP, three different chain-specific UBDs (M1-TUBE and K63-TUBE from Lifesensors and K48-UIM from Peter's Laboratory (18)) were employed to enrich specific M1-, K48-, and K63-modified substrates in the mouse liver (Fig. 4*A*). The enriched samples were then separated and verified through SDS-PAGE and immunoblotting with ubiquitin chain-specific antibodies. The K48 signal was most prominent, followed by the K63 signal, while the M1 signal was relatively weaker, which was consistent with the proportion of the respective ubiquitin chains present in organisms (41) (Fig. 4, *B*–*D*). Due to the low abundance, the signals of M1- and K63-linked chains in the input samples were not readily apparent; however, specific signals could be observed in the eluted samples after enrichment, indicating the effectiveness of enrichment. Moreover, the signals of M1- and K63-linked chains in the DRUSP group were significantly greater than those in the Control group (Fig. 4, *B* and *D*). Because of the high abundance of K48-linked chains, obvious signals can be detected in the input samples (Fig. 4*C*). Under equal loading amounts, the K48 signals in the DRUSP method were significantly greater than those obtained using the Control method. This indicated that denaturation enables more stable K48 ubiquitin chains to be obtained. After enrichment, the levels of K48 in the eluate were significantly greater within the DRUSP (Fig. 4*C*).

To make a more accurate quantitative comparison between these two methods, eight ubiquitin chains were subjected to targeted analysis using the SRM method (detailed information about the SRM method can be obtained in Supplemental Table S1). In terms of M1-specific UBD enrichment, we successfully identified the characteristic peptide for the M1 chain in the DRUSP dataset and found that its signal intensity was more than twice as high as that obtained from the traditional method (Fig. 4*E*). The levels of other ubiquitin chains, including K11, K48, and K63, also increased to varying degrees. In the K48-specific UBD-enriched samples, the characteristic peptide of K48 was also successfully identified, with a signal strength that increased by more than 3-fold. Additionally, other ubiquitin chains, such as K11, K27, and K33, were also identifiable (Fig. 4*F*). Similarly, in the K63-TUBE–enriched dataset, the signal strength of the characteristic peptide of K63 increased by more than 6-fold, and other chains, such as K11, K27, and even low-abundance M1, could also be detected (Fig. 4*G*). The present study demonstrated that DRUSP provided better results because it allowed for more adequate extraction. Both low-abundance M1 and K63 and high-abundance K48 can be extracted more stably and efficiently enriched by certain UBDs, which makes it possible to conduct more in-depth research on different types of ubiquitin chain proteomics.

### DRUSP-Based Ubiquitinomics Research Exhibited High-Performance Reproducibility and Quantitative Accuracy

**Fig. 5:**
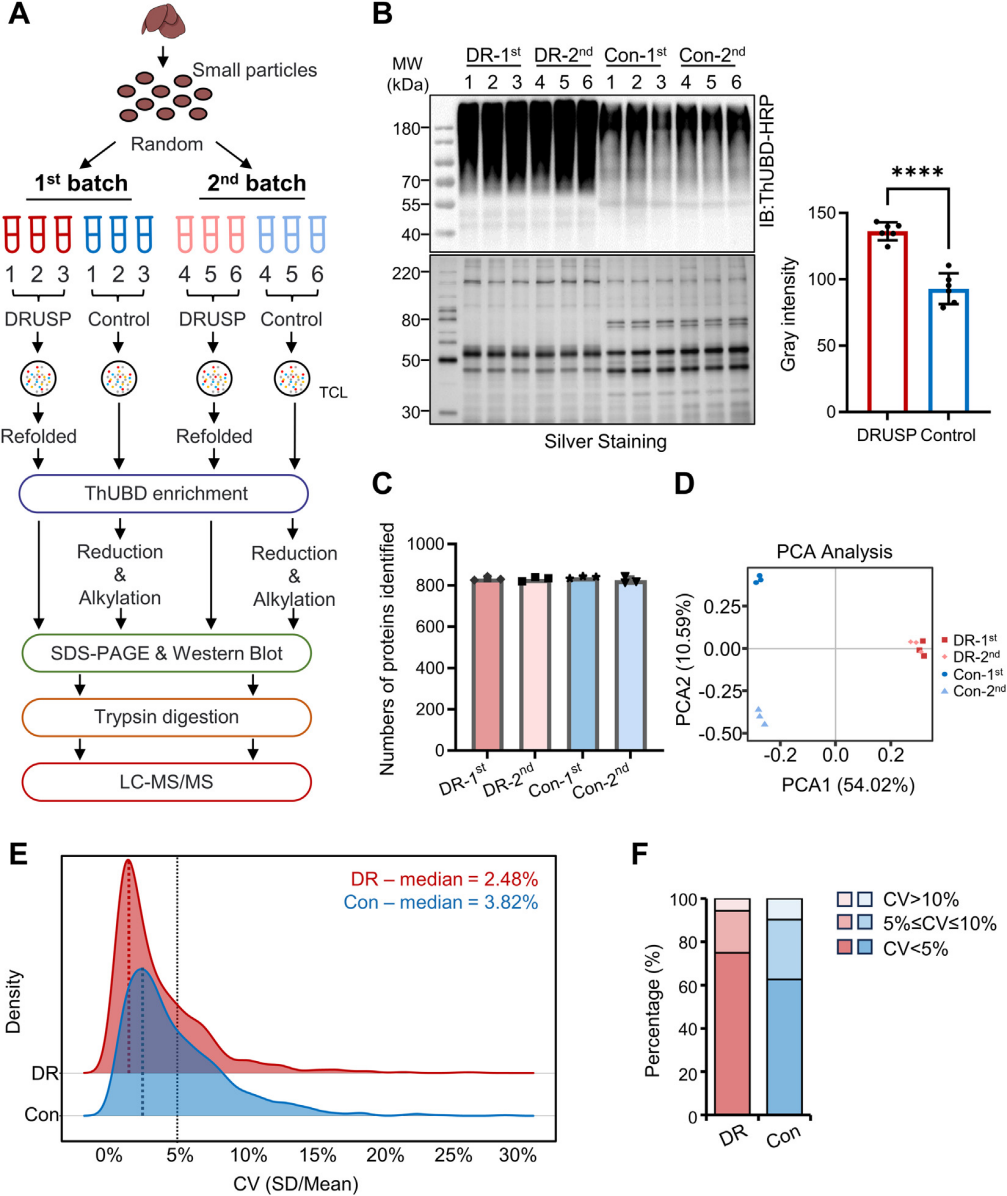
**Comparison of the stability and reproducibility of ubiquitinated proteins enriched by DRUSP and Control methods**. *A*, workflow for evaluating the stability and reproducibility of the two methods. Mouse livers were cut into pieces and divided into 12 parts of the same weight in liquid nitrogen. Each of the six samples was used for the same batch of experiments, three samples were used for each condition, and the experiments between batches were strictly carried out under parallel conditions. *B*, immunoblot analysis of two batches of enriched ubiquitin signals under two conditions, with silver staining as a loading control; the bar chart showed the *gray* values of DRUSP and Control in the Western blot, and the graphs indicate the mean ± S.D. (n = 6) derived from separate lanes in each group. *C*, numbers of identified proteins in two different batches under two conditions (batch 1, DRUSP - *heavy red*, batch 2 of DRUSP - *light red*, batch 1 of Control - *heavy blue*; and batch 2 of Control - *light blue*). *D*, principal component analysis (PCA) of two batches of ubiquitinome data between the DRUSP and Control groups is shown. The six groups of batches 1 and 2 under DRUSP clustered together, while the Control groups showed obvious separation between different batches. *E*, the density distribution of CV values (SD/Mean) is shown. The median CV was approximately 2.48% under DRUSP treatment and 3.82% under Control treatment. *F*, the bar chart shows the distribution of CV values below 5%, 5%-10%, and more than 10%. The CVs below 5% for DRUSP accounted for 78%, while they accounted for only 60% for the Control group.

To assess whether our improved DRUSP method has greater reproducibility and quantitative accuracy than traditional methods, we designed a quantitative workflow with different batches (Fig. 5*A*). The mouse liver was fragmented into small particles in liquid nitrogen and randomly divided into 12 equal-weight parts for assessment *via* the DRUSP and traditional methods (Fig. 5*A*). The results demonstrated that there were more consistent ubiquitin signals under DRUSP than under the Control method, which exhibited fewer fluctuations (Fig. 5*B*). Approximately 800 proteins were identified in every batch *via* the two methods (Fig. 5*C* and Supplemental Table S3). Principal component analysis of the quantitative protein information showed that the two methods could be used for clear distinction. The traditional method displayed distinct clustering based on two batches, while the DRUSP method formed one cohesive group (Fig. 5*D*). Similarly, hierarchical cluster analysis revealed that these proteins could be clustered into two groups but could be distinctly separated by batches under Control conditions, whereas this distinction was not observed under DRUSP conditions (Supplemental Fig. S6*A*). Both intra- and inter-batch correlations were stronger for DRUSP than for the control method (Supplemental Fig. S6*B*). A total of 78.8% of the proteins were quantified in the DRUSP datasets, while 72.6% were quantified in the Control datasets, confirming the high reproducibility and robustness of the DRUSP method (Supplemental Fig. S6*C*).

**Fig. 6:**
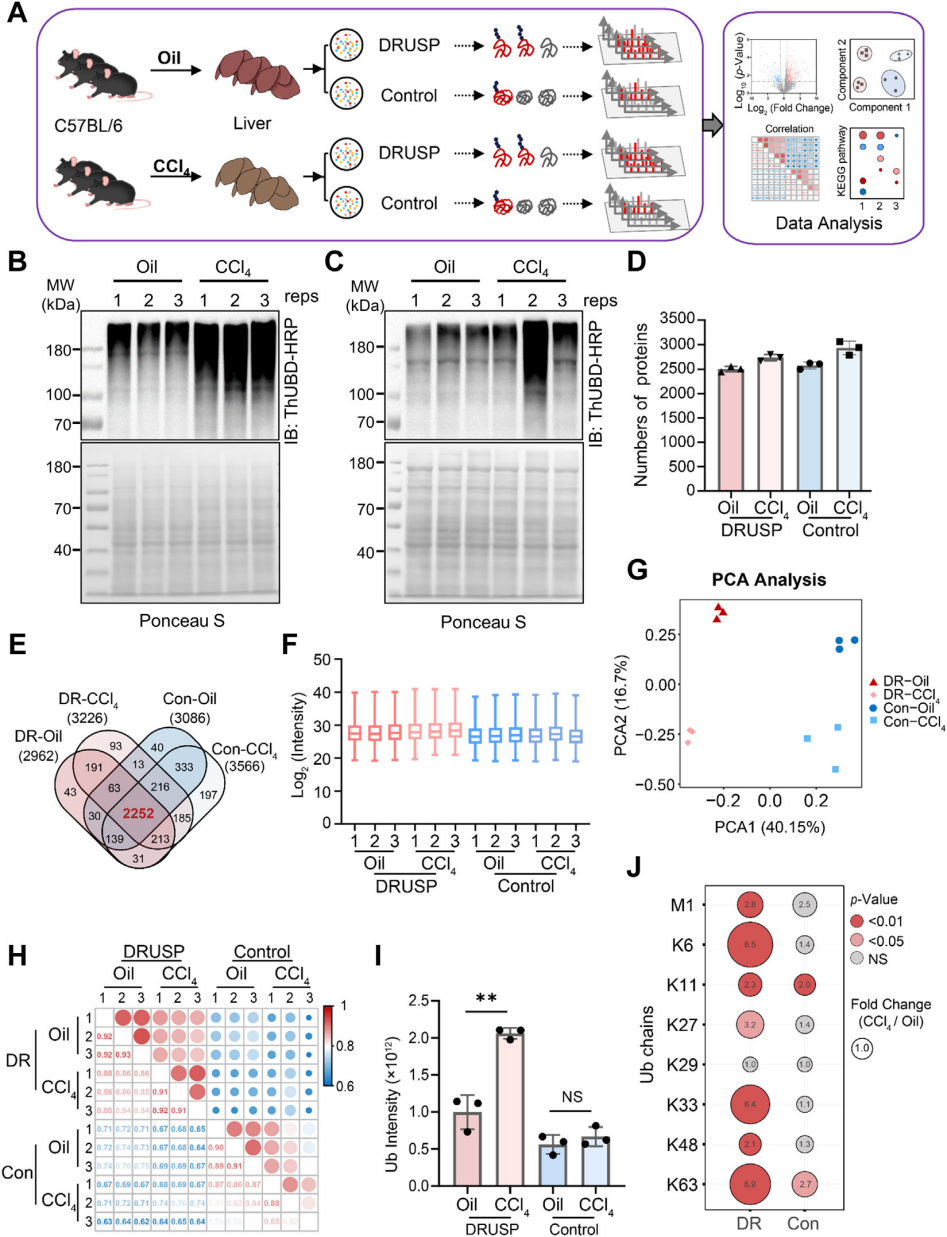
**DRUSP was applied for global ubiquitination enrichment in early hepatic fibrosis**. *A*, schematic of an early liver fibrosis mouse model. C57BL/6 mice were treated with oil or CCl_4_ and euthanized after 4 weeks. The ubiquitinated proteins were enriched through DRUSP and Control methods, digested with trypsin, and analyzed by LC-MS/MS. *B* and *C*, immunoblot analysis of global ubiquitination changes in early hepatic fibrosis tissues extracted from mouse livers subjected to the DRUSP (*B*) and Control (*C*) methods. Ponceau S was used as a loading control. *D*, numbers of proteins identified in the oil- and CCl_4_-treated ubiquitinome groups according to the DRUSP and Control methods. *E*, Venn diagram shows the proteins that overlapped between the four ubiquitinome groups. *F*, the box diagram shows the overall distribution of the log_2_ intensity of the ubiquitinome datasets. *G*, principal component analysis (PCA) of four different ubiquitinome groups. *H*, the correlation coefficients of the different groups are presented in *red* (high-positive correlation) and *blue* (lower correlation). *I*, the absolute ubiquitin signal in each group. The data represent the average of three independent biological replicates and were analyzed using a two-tailed paired *t* test. The graphs show the means ± S.D. (n = 3) derived from three independent experiments. ∗∗*p*-value <0.01; NS, not significant. *J*, dot plot of the changes in the levels of eight ubiquitin chains in the ubiquitinome group before and after CCl_4_ treatment in both the DRUSP and Control groups. The size of the dot is based on the fold change, and the color of the dot indicates the significance.

Additionally, the median coefficient of variation for the overall data was smaller in the DRUSP dataset, with 75% decreasing below 5% and only 62.6% for traditional conditions (Fig. 5*E*). Differential protein screening was performed between different batches of the two methods. Only six upregulated proteins and one downregulated protein were screened out under DRUSP, while 97 upregulated proteins and 15 downregulated proteins were screened out under the Control method (Fig. 6, *D* and *E*). The SD of the log_2_ ratio distribution between the two batches of DRUSP was 0.34, while it increased to 0.7 in the Control method (Supplemental Fig. S6, *F* and *G*). In summary, we demonstrated the advantages of high quantitative accuracy and a low coefficient of variation using the DRUSP method for ubiquitinomics research.

### The DRUSP Method Revealed More Accurate Quantification of Ubiquitin Signalling Changes in Early Hepatic Fibrosis

Previous studies have shown that the development of liver fibrosis is associated with ubiquitination, but further in-depth and systematic research is needed (42, 43, 44, 45, 46). To further validate the application of DRUSP in practical scenarios, we selected an early-stage mouse liver fibrosis model to compare differences in the ubiquitinome. An early-stage liver fibrosis mouse model was established by treating C57BL/6 mice with CCl_4_ for 4 weeks. Successful establishment of the model was confirmed through HE staining, Sirius scarlet staining, and serum ALT level analysis (Supplemental Fig. S7, *A*–*C*). Subsequently, the negative control and fibrotic mouse liver tissues were lysed and enriched in parallel through two methods to explore comprehensive alterations in ubiquitination (Fig. 6*A*). Western blot analysis revealed that the global ubiquitination level increased to varying extents in early-stage liver fibrosis (Fig. 6*B*). However, there was a significant difference between the two methods, with more consistent and accumulated ubiquitin signals observed under the DRUSP method. Conversely, the signals of ubiquitination exhibited notable fluctuations under the Control condition (Fig. 6, *B* and *C*). We previously demonstrated that ThUBD-HRP can be utilized for detecting ubiquitin signals in FFPE samples by immunohistochemical staining (29). Therefore, we employed a ThUBD-HRP probe for immunohistochemical verification and revealed an obvious increase in ubiquitination *in situ*. This finding provided preliminary validation for our hypothesis that the DRUSP method could show a more realistic state in which ubiquitin signals were significantly accumulated in the fibrotic liver (Supplemental Fig. S7*D*). Silver staining clearly revealed more smeared lines in the high-molecular-weight region after treatment with the DRUSP method than the Control method (Supplemental Fig. S7, *E* and *F*).

To further verify this difference, LC-MS/MS analysis of these samples was conducted. We identified an average of approximately 3000 proteins in each group (Fig. 6*D*), of which 2252 overlapped (Fig. 6*E* and Supplemental Table S4). Although there was minimal variation, there was a higher median value for the DRUSP method at the overall intensity (Fig. 6*F*). Principal component analysis showed significant differences between the four groups, and the degree of dispersion between groups under the control method was greater (Fig. 6*G*). Compared with the Control method, the DRUSP method had a greater correlation (Fig. 6*H*). The total ubiquitin intensity significantly increased two-fold between the CCl_4_ group and the oil group under the DRUSP treatment but did not significantly change under the Control method (Fig. 6*I*). For eight ubiquitin chains, seven chains, excluding K29, exhibited an increasing trend according to the DRUSP method, indicating greater statistical significance (Fig. 6*J*, left). In contrast, only K11 and K63 chains showed a significant increase when quantified using the Control method (Fig. 6*J*, right). DRUSP demonstrated superior accuracy and robustness in quantification compared to the traditional method for ubiquitinomics research.

### DRUSP Method Revealed a Broader Global Landscape of Ubiquitination Disorders in Early Hepatic Fibrosis

**Fig. 7:**
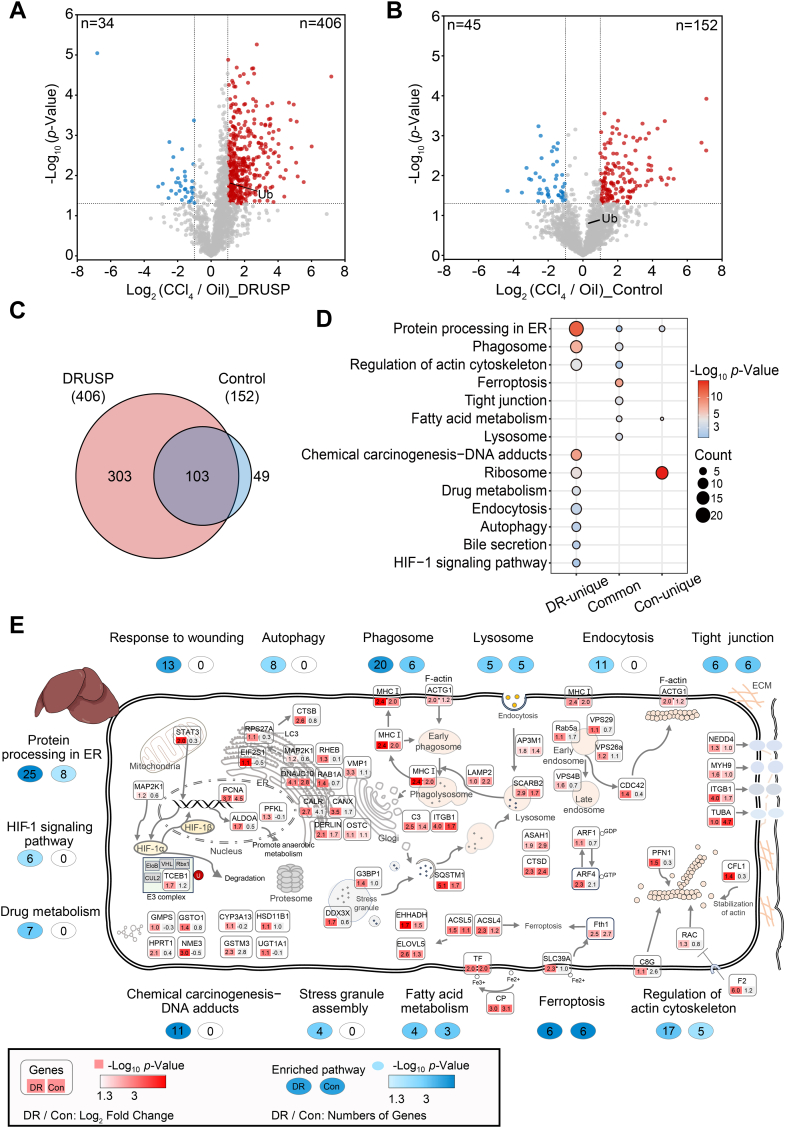
**Deep ubiquitinomic profiling *via* DRUSP method revealed ubiquitinome disorders in early hepatic fibrosis models**. *A*, volcano plot of the ubiquitinome distribution between CCl_4_ and oil treatment derived from the DRUSP dataset is shown. Significantly upregulated proteins with *p*-value <0.05 are colored (Log_2_ FC −1.0 ≤ x ≤ 1.0 in *gray*, x > 1.0 in *red*, and x < −1.0 in *blue*). *B*, volcano plot of the ubiquitinome distribution between CCl_4_ and oil treatment derived from the Control dataset is shown. *C*, the overlap of upregulated proteins identified by the DRUSP (n = 406) and Control (n = 152) methods, respectively. *D*, dot plot shows the KEGG pathways of the unique upregulated ubiquitinated proteins in the DRUSP dataset, the overlapping proteins, and the unique proteins in the Control dataset. The size of the dot is based on the number of genes enriched, and the color of the dot indicates the significance of the enrichment. *E*, biological landscape of partially upregulated ubiquitinated proteins in an early mouse hepatic fibrosis model. The diagram summarizes the upregulated ubiquitinated proteins identified by the two methods. *Red*, *p*-value <0.05; *gray*, not significant. The number of genes enriched in the DRUSP and Control groups is shown, respectively. *Blue* represents a significant *p*-value, and *white* represents no enrichment.

With the DRUSP method, 406 upregulated ubiquitinated proteins were identified, while only 152 upregulated proteins were identified through the Control method (Fig. 7, *A* and *B*, Supplemental Tables S5, and S6). Among these proteins, 103 overlapped, while 303 unique proteins were identified *via* the DRUSP method (Fig. 7*C*). Compared to Control methods, the DRUSP method can more effectively screen potentially upregulated ubiquitinated proteins related to liver fibrosis. We conducted functional analysis on both unique and overlapped proteins from panel C. KEGG pathway analysis revealed that common proteins were primarily involved in ER protein processing, phagosome regulation, actin skeleton regulation, ferroptosis, tight junctions, and lysosomes (Fig. 7*D*, middle). Additionally, the uniquely altered proteins from the DRUSP dataset also showed additional involvement in chemical carcinogen-DNA adducts, drug metabolism, endocytosis, autophagy, and the HIF-1 signaling pathway (Fig. 7*D*, left). The commonly upregulated proteins were mainly found in the collagen-containing extracellular matrix, basement membrane, endoplasmic reticulum, peroxisomes, and other cellular locations (Supplemental Fig. S8*A*). These proteins were involved in various biological processes, such as protein localization to vacuoles, response to methanol, acute-phase response, iron ion transport, cell-matrix adhesion, endocytosis regulation, and metabolism (Supplemental Fig. S8*C*). Through the DRUSP method, unique proteins, such as those in the Golgi membrane, smooth endoplasmic reticulum, myelin sheath, and cell cortex, were found to exhibit specific localization (Supplemental Fig. S8*B*). These proteins were involved in a broader range of biological processes, including the modification of synaptic structures, Golgi organization, response to endoplasmic reticulum stress, regulation of response to wounding, intracellular protein transport, and protein maturation (Supplemental Fig. S8*D* and Supplemental Table S7). The DRUSP method enriches a wider range of comprehensive pathways.

Furthermore, the significantly upregulated proteins in the enrichment pathway were selected and visually represented more intuitively (Fig. 7*E*). Ubiquitination plays a widespread role in various physiological processes within the cell, including the mitochondria, nucleus, endoplasmic reticulum, Golgi apparatus, lysosome, and other cellular organelles. Currently, although liver fibrosis research is attractive, the relationship between liver fibrosis and ubiquitination remains incomplete. In our study, we confirmed the global upregulation of ubiquitination during early-stage liver fibrosis, which is consistent with the changes in ubiquitination levels observed in CCl_4_-induced liver fibrosis in ^bio^Ub transgenic mice by *Teresa C. Delgado et al* (44). Our finding based on the DRUSP that ubiquitination involves multiple modules of cellular life activities provided a more comprehensive landscape than traditional methods.

In our dataset, we observed a greater number of differentially ubiquitinated proteins using the DRUSP method than using the Control approach. Specifically, our results indicated a substantial increase in the ubiquitination levels of signaling transcription factors, specifically signal transducer and activator of transcription 3 (STAT3), with the DRUSP method but not with the Control method (Fig. 7*E*). STAT3 has critical functions in various cellular processes, such as cell survival and proliferation, and is a key upstream regulator of the HIF-1 signaling pathway in hypoxic metabolism (47, 48). Its presence is essential for HIF-1α activity and downstream signal transduction. Extensive experiments have shown that inhibiting STAT3 activation can effectively treat acute liver injury and liver fibrosis, making STAT3 an attractive target for therapeutic strategies (49). Our novel observation provides new insights into the role of STAT3 ubiquitination in the progression of liver fibrosis within the context of the HIF-1 pathway, offering potential directions for targeted interventions.

Additionally, we observed increased ubiquitination of the acyl-CoA synthetase long-chain family member 4 (ACSL4) in our dataset. Notably, its ubiquitination level exhibited a 2.3-fold increase under the DRUSP method compared to a 1.2-fold increase under the traditional method (Fig. 7*E*). ACSL4 is an essential molecule that promotes iron-induced cell death through the accumulation of oxidized phospholipids in cell membranes (50). Previous studies have highlighted the critical role of ferroptosis in liver fibrosis progression (43, 51). Our findings provide valuable insights into ferroptosis and liver fibrosis.

Proteins identified through the DRUSP method were also identified using the traditional (Control) approach. However, their ubiquitination levels either exhibited no significant upregulation or lacked significance altogether. These proteins include STAT3, MAP2K1, ALDOA (associated with hypoxic metabolism), CTSB (linked to the autophagy pathway strongly connected to liver fibrosis), vacuolar protein sorting family proteins (associated with the endocytosis pathway), and the actin skeleton-related proteins CDC42, PFN1, and CFL1 (key molecules strongly associated with liver fibrosis) (48, 52, 53). Similarly, the ubiquitination levels of proteins such as CALR, CANX (related to endoplasmic reticulum protein folding), G3BP1 (related to stress particle formation), ARF4 (important molecules related to cancer processes), and GSTM3 (metabolites related to chemical carcinogenesis) were upregulated, but the differences were not significant (54, 55, 56). These proteins play vital roles in liver fibrosis, but their ubiquitination and functional implications have not received much attention. Therefore, our newly developed DRUSP method offers broader perspectives and directions for investigating the ubiquitinome in relation to liver fibrosis.

## Discussion

In this study, the proteomic sample preparation process was organically combined with the traditional UBD enrichment process, addressing existing ubiquitinomics challenges. By leveraging the stability and folding properties of ubiquitin molecules, we demonstrated that denatured ubiquitin chains can be restored to their correct spatial structure and unbiasedly recognized by ThUBD. Using the traditional UBD approach, we proposed an innovative method called DRUSP, which overcomes common issues encountered in enriching ubiquitinated proteins under native conditions. First, our method ensured more adequate extraction under denaturing conditions. Through extraction, most proteins can be fully unfolded, effectively blocking enzyme active sites and protecting ubiquitinated proteins from degradation. Second, the DRUSP method can disrupt numerous interacting proteins to reduce background noise. Third, DRUSP has broader applicability in the presence of denaturing agents such as urea and SDS. It can be applied to various biological samples, including fibrotic liver samples with altered hardness and texture and FFPE samples with severe crosslinking, which are typically challenging to extract from. Finally, compared to traditional methods, our approach achieved greater efficiency and reproducibility in enriching ubiquitinated proteins. Furthermore, we demonstrated that the DRUSP method was equally effective for different materials, including chain-specific UBDs and unbiased ThUBD.

We believe that the DRUSP method possesses advantages in terms of efficient enrichment, reproducibility, and low variability. However, there is still significant room for improvement and optimization. By combining the specificity of ubiquitin chains or K-ε-GG antibody enrichment materials with DRUSP, this approach has the potential to become a core technology strategy in ubiquitinomics and to play a more substantial role. Given the versatility of this method, we hope to apply it to more specific biological models in the future, such as advanced liver cancer, neurodegenerative diseases, cerebral amyloid vascular disease, and other conditions characterized by severe pathological changes. Using Control (traditional) methods hinders protein extraction and poses challenges to the study of mechanisms or exploration of global changes in these biological models; hence, we aim to facilitate their investigation. Additionally, we anticipate that this method can be utilized for crosstalk analysis with different posttranslational modifications and for exploring changes in PTMs on the same protein. This will provide an improved experimental framework for studying posttranslational modifications. To our knowledge, our work is pioneering in proposing refolded proteins after denatured extraction and utilizing ThUBD for enriching ubiquitinated proteins. Besides ubiquitinomics research, DRUSP could be applied with biochemistry analysis of ubiqutination signal for certain target protein by WB. Furthermore, we have demonstrated its strong advantages across various aspects; therefore, this method may emerge as a powerful and widely adopted strategy at the protein level for enriching ubiquitinated proteins.

When the DRUSP method was applied to biological or clinical investigations, it showed powerful performance for ubiquitinome identification and quantification. Ubiquitination plays a crucial role in all processes of life, and a thorough and comprehensive understanding of protein ubiquitination is essential. Compared to Control methods, the DRUSP method appears capable of facilitating exploration of previously unknown and more comprehensive aspects of ubiquitination. In our study, we applied the DRUSP method to an actual early-stage liver fibrosis model. The results confirmed that ubiquitin was upregulated after liver fibrosis, which is consistent with the findings of previous studies. However, under Control methods, the upregulation of ubiquitination is only an individual event. Ubiquitination is closely related to the pathogenesis of liver fibrosis, as confirmed in previous studies (46, 57, 58). Therefore, the use of the DRUSP method can provide a more comprehensive global perspective on the changes in ubiquitination that occur more reliably during disease. Moreover, our new method can overcome the defects of traditional methods, such as inaccurate quantification and easy distortion of ubiquitination signals. Moreover, it can offer a novel direction for investigating the pathological mechanisms underlying disease and help us find more effective target molecules for disease diagnosis and treatment.

## Data Availability

The mass spectrometry proteomics data have been deposited to the ProteomeXchange Consortium (https://proteomecentral.proteomexchange.org) *via* the iProX partner repository (59, 60) with the dataset identifier PXD055428 or IPX0008016000. Annotated spectral files were uploaded to the MS-viewer (61). The search key for the ubiquitinated peptides is *nbor6em x 8h*. The search key for assessing quantitative stability and reproducibility is *ldpvudrlr2*. The search key for ubiquitinated proteins of mouse fibrosis liver is *zobihveoh5*.

## Supplemental data

This article contains supplemental data. Supplemental Information, including Supplemental Methods, eight figures, and seven tables, can be found online (16, 22, 28, 29, 31, 62).

## Conflicts of Interests

The authors declare no competing interests.
