# Supplementary material for: Super Enhanced Purification of Denatured-Refolded Ubiquitinated Proteins by ThUBD Revealed Ubiquitinome Dysfunction in Liver Fibrosis
Source: Mol Cell Proteomics. 2024 Oct 2;23(11):100852. doi: 10.1016/j.mcpro.2024.100852 (PMC11584597; doi:10.1016/j.mcpro.2024.100852)
Supplement: Supplement Information [file mmc1.docx]

# Supplement Methods

**CCl_4_ induced early-stage liver fibrosis model establishment**

To establish an early hepatic fibrosis model, all mice were intraperitoneally injected with CCl_4_ (200 μL per mouse, dissolved in olive oil at a ratio of 1: 7) every 2 days for 4 weeks and a vehicle was used as a control ^[31]^. Their serum and liver samples were collected for subsequent analysis. Serum ALT activity was detected using automatic biochemical analyzer (HITACHI).

**FFPE mouse liver samples**

The liver samples were promptly excised following the sacrifice, followed by fixation in formalin and paraffin embedding. Ten-micrometer-thick sections were sliced from a single liver FFPE tissue block using a microtome, and mounted onto glass slides.

**ThUBD-HRP probe and affinity matrix preparation**

An artificial tandem hybrid ubiquitin-binding domain (ThUBD) material was used in this study, which is highly enriched in affinity and comprises a glutathione S-transferase (GST) tag. The specific method of plasmid construction is described previously ^[16]^. The ThUBD was expressed in *Escherichia coli* BL21 (DE3) cells, which was induced with 1 mM isopropyl 1-thio-β-D-galactopyranoside (IPTG) for 14 h at 16 °C. The harvested cells were lysed by sonication in lysis buffer (20 mM Tris-HCl pH 7.5, 150 mM NaCl, 5% glycerol, 1 mM DTT). GST-tagged ThUBD were purified from cell lysates using glutathione-Sepharose (GSH) 4B beads (Sangon Biotech). The purified ThUBD proteins were competitively eluted by reduced glutathione and then displaced into PBS buffer before being coupled to NHS-activated Sepharose (Cytiva) following the manufacturer’s instructions. The protein concentration was measured through SDS-PAGE, and the coupling was performed according to 1 μL NHS beads to 5 μg ThUBD proteins. The UBD-conjugated agarose (ThUBD affinity matrix) was stored at -20 °C in PBS supplemented with 50% glycerol.

For HRP labeling, the concentration of freshly purified ThUBD was adjusted to 2 μg / μL and then incubated with EZ-link maleimide activated horseradish peroxidase (HRP) at a molecular ratio of 1: 4 ^[28]^. The labeling experiment was performed as described previously and concentration of the eluted protein was determined through Coomassie Brilliant Blue staining by SDS-PAGE. The labeled protein was stored in 20% glycerol at -30 °C.

**Ubiquitinated peptides enriched by K-ε-GG antibody**

Lyophilized peptides from DRUSP and Control were re-suspended in immunoaffinity purification (IAP) buffer (50 mM MOPS, pH 7.2, 10 mM Na_2_HPO_4_, 50 mM NaCl) and sonicated for 10 min. Di-Gly remnant containing peptides were enriched using the PTMScan® Ubiquitin Remnant Motif (K-ɛ-GG) Kit (Cell Signaling Technology (CST)) ^[22]^. First, antibodies were cross-linking to beads according to Udeshi et al ^[62]^. Second, the peptide mixture was incubated with K-ε-GG antibody-crosslinked beads for 2 h at 4 °C. After incubation and gentle agitation, beads were washed twice with IAP buffer and twice with cold ddH_2_O. The bonded peptides were eluted four times with 20 μL of 0.15% TFA, and the eluted sample was desalted using an in-house packed C18 StageTip prior to LC-MS/MS analysis as follows: (a) Methanol (MeOH) and acetonitrile (ACN) were successively used in pretreatment; (b) Buffer B (80% ACN / 0.1% FA) and Buffer A (1% ACN / 0.1% FA) were used to balance the column bed; (c) acidified to pH < 3 by addition of FA and loaded samples twice; (e) washing with Buffer A; (f) elution with 50% ACN / 0.1% FA. The desalted samples were dried by vacuum centrifugation (Thermo Scientific), and the peptides were frozen at -80 °C.

**Immunohistochemistry (IHC)**

For detection of ubiquitination signals *in situ* level, samples were fixed with 4% paraformaldehyde and cut into slices. After deparaffinization and rehydration, antigen retrieval was achieved by heating in a pressure cooker for 10 min in 10mM of sodiumcitrate (pH 6.0). Peroxidase activity was blocked by incubation in 3% H_2_O_2_ for 30 min at 37 ℃. Sections were rinsed three times in PBS and incubated in 5% BSA for 30 min. After the removal of the blocking solution, slides were placed into a humidified chamber and incubated for 2 h with ThUBD-HRP (diluted at 1: 1000) in blocking buffer (5% BSA in PBS) at 4 °C ^[29]^. After washing, the signals were directly visualized through the reaction between its conjugated HRP and the DAB (3,3′-diaminobenzidine tetrahydrochloride) substrate. The sections were sealed with neutral gum and observed under a light microscope. Images were acquired using an inverted microscope (OLTMPVS CKX41).

For validation of hepatic fibrosis model, paraffin-embedded liver samples were sectioned and stained with haematoxylin-eosin (HE), Masson's trichrome and Sirius red according to manufacture protocols. The positive area was quantified using the ImageJ 1.48v software (National Institutes of Health).

**LC-MS/MS sample preparation and analysis**

For in-gel digestion, samples were separated on 10% SDS-PAGE and cut into five different slices based on the molecular weight and protein abundance. Coomassie Brilliant Blue was removed by decolorization solution (30% ACN / 35 mM ammonium bicarbonate, pH 7 - 8), dehydrated, solidified with acetonitrile, and digested with trypsin overnight. Then the tryptic peptides were extracted from gels with extraction buffer (5% FA / 50% ACN) and centrifuged at 17,000 ×g. Finally, samples were dried using a vacuum dryer before LC-MS/MS analysis. For in-solution digesting, proteins were digested with trypsin at a ratio of 1: 50. Then peptides were desalted using an in-house packed C18 StageTip prior to LC-MS/MS analysis as described previously.

For early-stage liver fibrosis detection, the resulting peptides were analyzed using an ultra-performance LC-MS/MS platform of hybrid LTQ-Orbitrap Fusion Lumos mass spectrometers (Thermo Fisher Scientific, USA). Peptides were separated on a 75 µm I.D. × 20 cm capillary column packed with 1.9 µm C18 reverse-phase fused-silica (Michrom Bioresources). The LC nonlinear gradient with 78 min ramped from 8% to 40% of mobile phase B (phase B: 0.1% FA in ACN, phase A: 0.1% FA / 1% ACN in water) at a nanoflow rate of 600 nL / min. The MS1 was detected with a mass range of 300 - 1,400 at a resolution of 60,000 at m/z 200. The automatic gain control (AGC) was set as 5 × 10^5^ and the maximum injection time (MIT) was 50 ms. For MS2 scan, the AGC was set at 1 × 10^4^ and the MIT was set at 80 ms. The dynamic range was set at 20 s to suppress repeated detection of the same ion peaks.

For the DRUSP method establishment, eluting peptides were analyzed using an LTQ-Orbitrap Velos mass spectrometer (Thermo Fisher Scientific). Peptides were separated on a 75 µm I.D. × 15 cm capillary column packed with 3 µm C18 reverse-phase fused-silica (Michrom Bioresources). The LC nonlinear gradient with 80 min ramped from 8% to 40% of mobile phase B (phase B: 0.1% FA in ACN, phase A: 0.1% FA / 1% ACN in water) at a nanoflow rate of 600 nL / min. The MS1 was detected with a mass range of 300 - 1600 at a resolution of 30,000 at m/z 400. The automatic gain control (AGC) was set as 1 × 10^6^ and the maximum injection time (MIT) was 150 ms. The MS2 was detected in data-dependent mode for the 20 most intense ions subjected to fragmentation in the linear ion trap (LTQ). For each scan, the AGC was set at 1 × 10^4^ and the MIT was set at 30 ms. The dynamic range was set at 25 s to suppress repeated detection of the same ion peaks.

**Data analysis**

All the raw files were searched by MaxQuant (version 2.1.4.0) against the Swiss-Prot reviewed mouse database (released on 2024_02_03, containing 17,201 entry proteins). Searching parameters consisted of full tryptic restriction and peptides were allowed up to two miss cleavages. The precursor mass tolerance was set at 20 ppm, and the fragment tolerance was set at 0.5 Da or 20 ppm. Carbamidomethylation of cysteine was specified as a fixed modification. Oxidation of methionine and protein N-terminal acetylation were assigned as variable modifications. For ubiquitinome identification, di-Glycine on lysine (+114.0429 Da) was also set as variable modification. Searching results were filtered to a 1% FDR at the proteins, peptides, and PSM levels using the target-decoy strategy. Peptides and proteins intensity were used for quantification.

Data analysis was primarily performed in the Perseus (version 2.0.3.0), the intensity of protein was transformed by log_2_, and each set of data was normalized based on the median values. For evaluation of reproducibility and accuracy, PCA analysis, correlation analysis, and coefficient of variation analysis were performed after median correction between batches. The differential protein screening was based on the protein with more than three quantitative values in each condition. In the CCl_4_ model group, only proteins with 4 quantitative values were used for subsequent analysis. Similarly, the analysis of differential proteins selected only 12 quantitative proteins as conditions for screening. Through the online website Metascape (<http://metascape.org/>) to carry out gene ontology (GO) analysis and KEGG pathway to obtain information about biological processes involved in differential proteins. Statistical analyses were performed using GraphPad Prism, version 8 (GraphPad). The drawing of a few graphics relied on an online platform for data analysis and visualization. (https://www.bioinformatics.com.cn (last accessed on 10 Nov 2023).

**SI figures and legends**


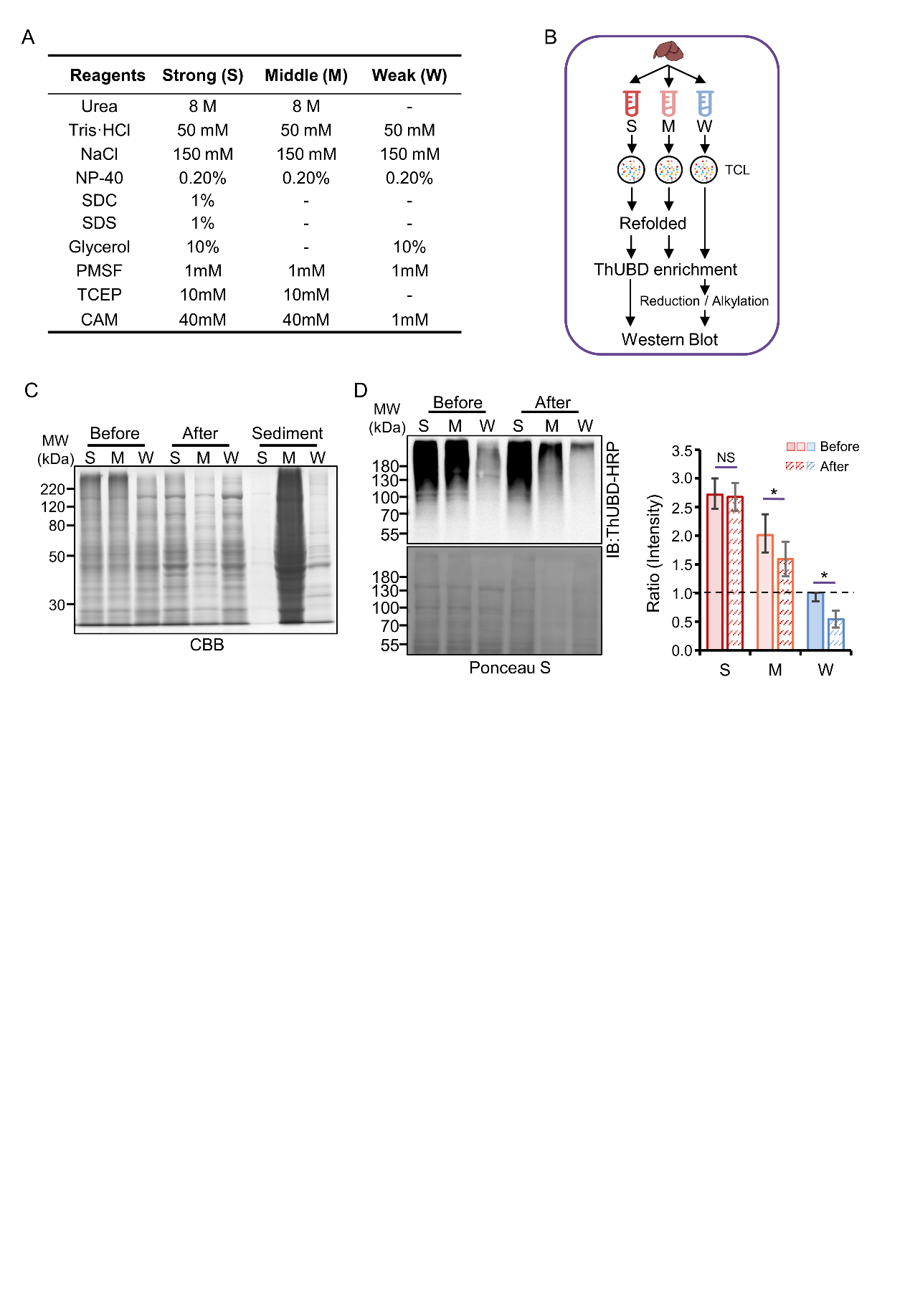


**Figure S1. Loss evaluation of the sample after denatured-refolded preparation.**

(A) Components of three types of lysis buffers, including strong (S), middle (M) and weak (W) ones. SDC, sodium dehydroeholate; SDS, Sodium dodecyl sulfate; NP-40, Nonidet P-40; PMSF, Phenylmethanesulfonyl fluoride; TCEP, Tris (2-carboxyethyl) phosphine; CAM, 2-Chloroacetamide.

(B) Workflow for extraction, refolded, purification, and detection using three buffer conditions. Equal amount of mouse livers were used for validation.

(C) Coomassie bright blue (CBB) staining of before and after refolded through three lysis conditions.

(D) For the verification and quantification of the ubiquitin signals before and after refolded, the ratio was 1 before weak replacement, and ponceau S was used as a loading control. The data represent the average of three independent experiments and were analyzed using a two-tailed paired t-test. Graphs indicated mean ± SD. * *p*-value < 0.05, ** < 0.01, *** < 0.001. NS, no significance.


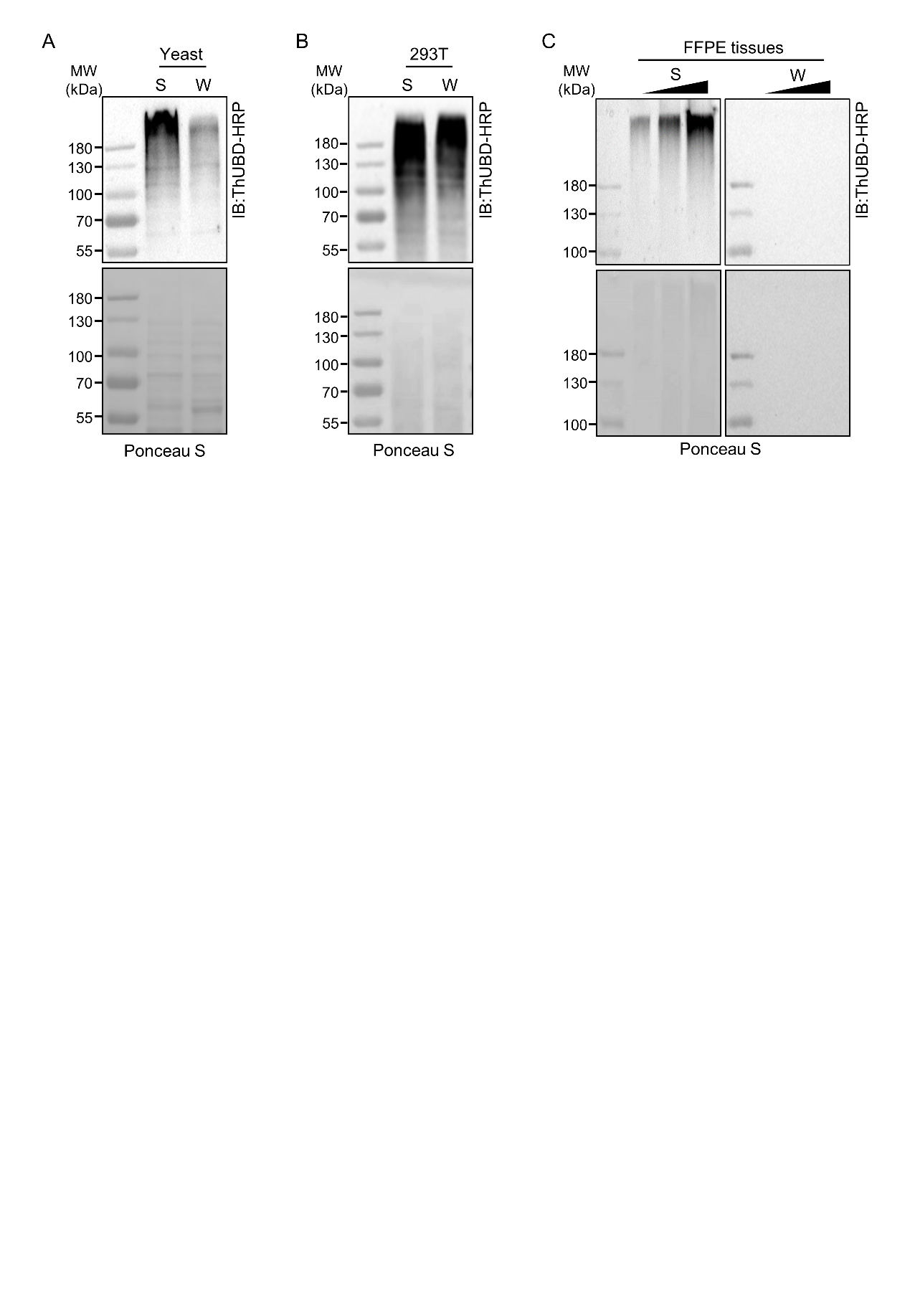


**Figure S2. Evaluation of the universality of DRUSP applied to different biological samples**

(A - C) Immunoblot analysis of ubiquitin signals under strong buffer and weak buffer with different samples, including yeast (A), mammalian cell 293T (B), and FFPE tissue (C), respectively.


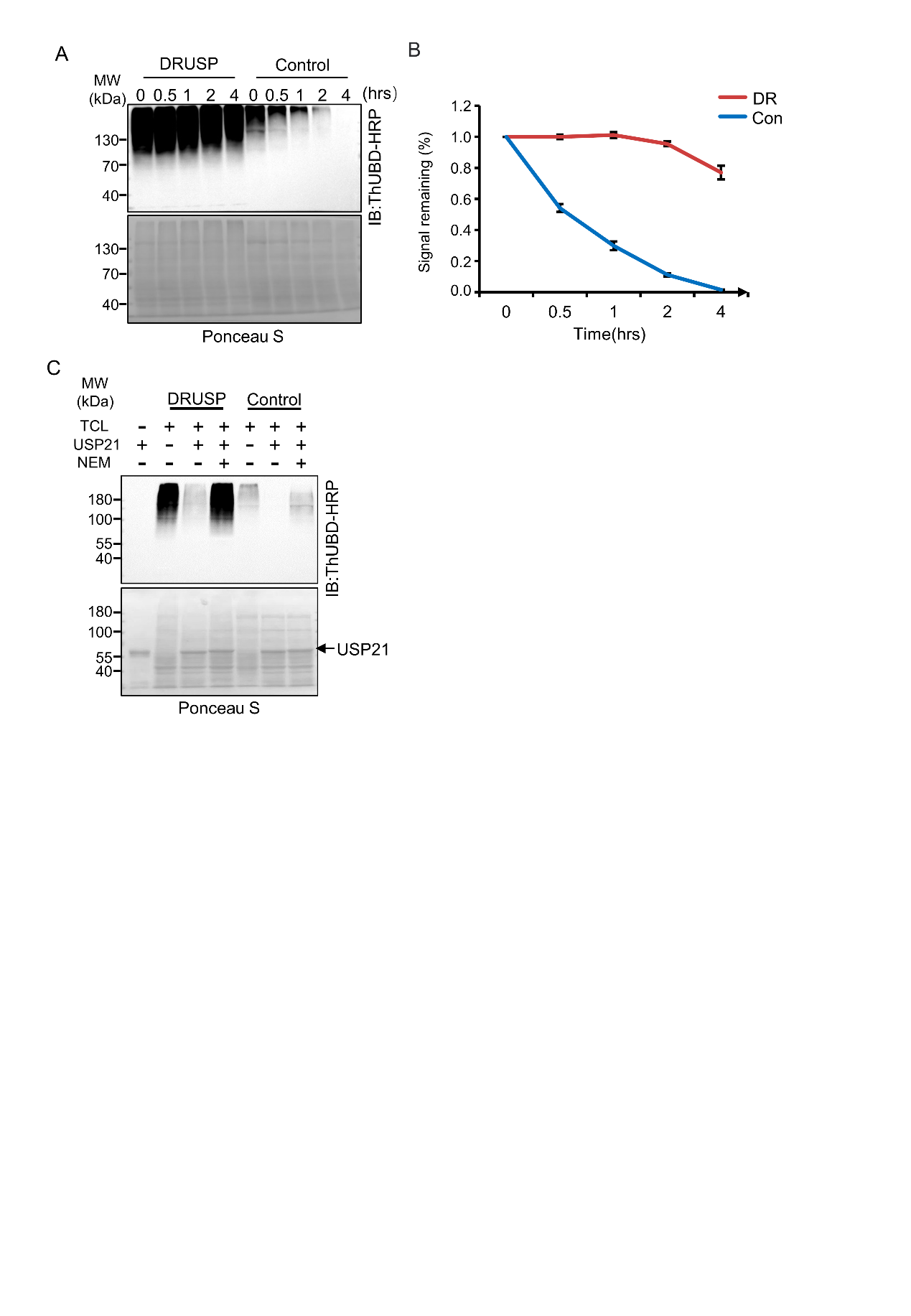


**Figure S3. The ubiquitin signal derived from DRUSP sample was more stable compared to that of Control method**

(A) The ubiquitin signal was more stable in the DRUSP samples. Western blotting was used to analyze the ubiquitination changes of denatured-refolded proteins and natively prepared proteins after being placed at 37 ℃ for 0, 0.5, 1, 2, 4 h.

(B) Graph of the changes of the ubiquitin signals between DRUSP and Control groups.

(C) Western blotting analysis of the changes of ubiquitin signal after USP21 non-specific cleavage.


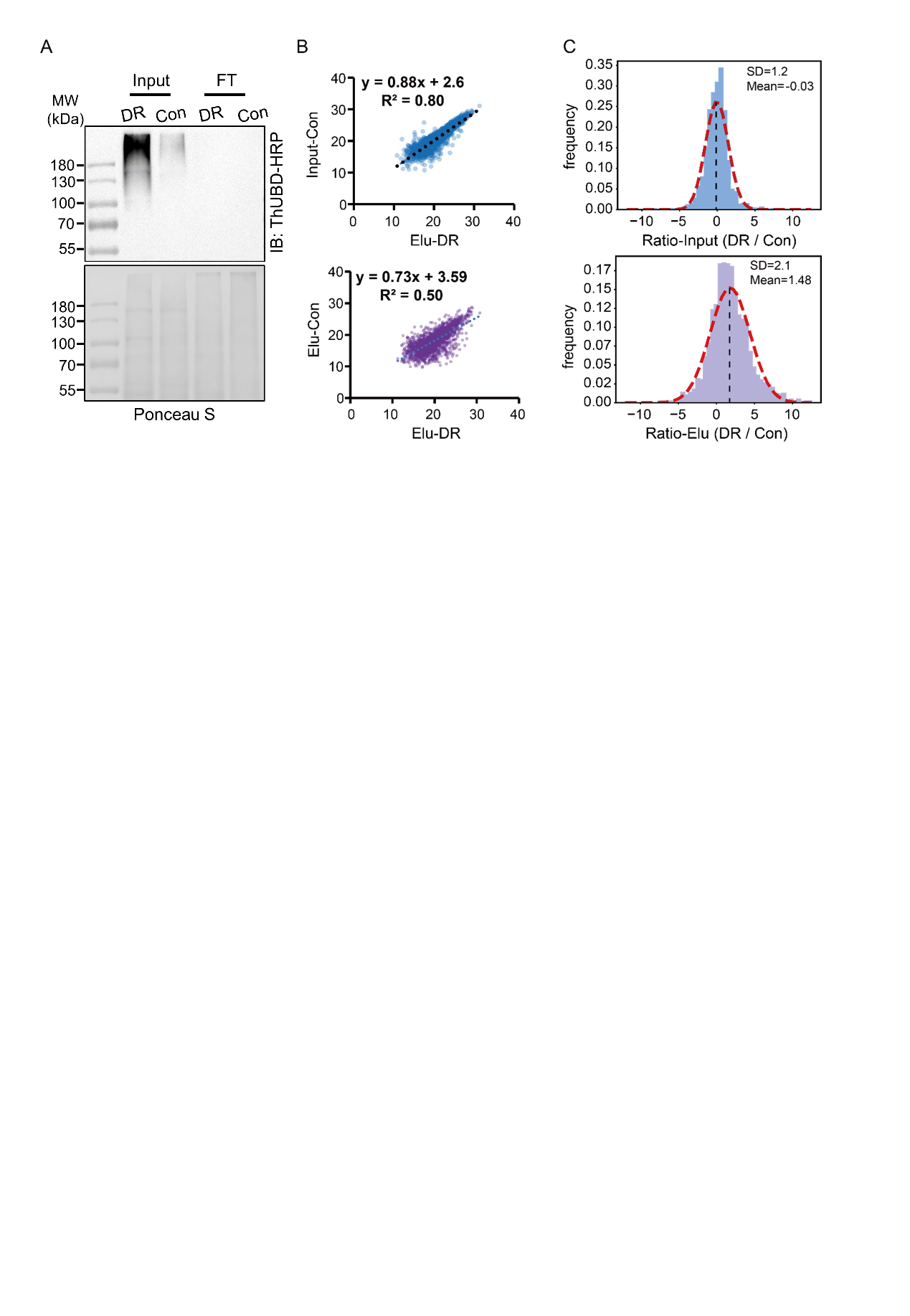


**Figure S4. Correlation and data distribution of proteome and ubiquitinome datasets derived from DRUSP and Control methods**

(A) The ubiquitin signals in input and FT were compared between DRUSP and Control groups.

(B) Correlation of log_2_ intensity of DRUSP and Control datasets in the whole proteome (up) and ubiquitinome (down) groups.

(C) Distribution of DRUSP to Control ratios in the whole proteome (up) and ubiquitinome (down) groups.


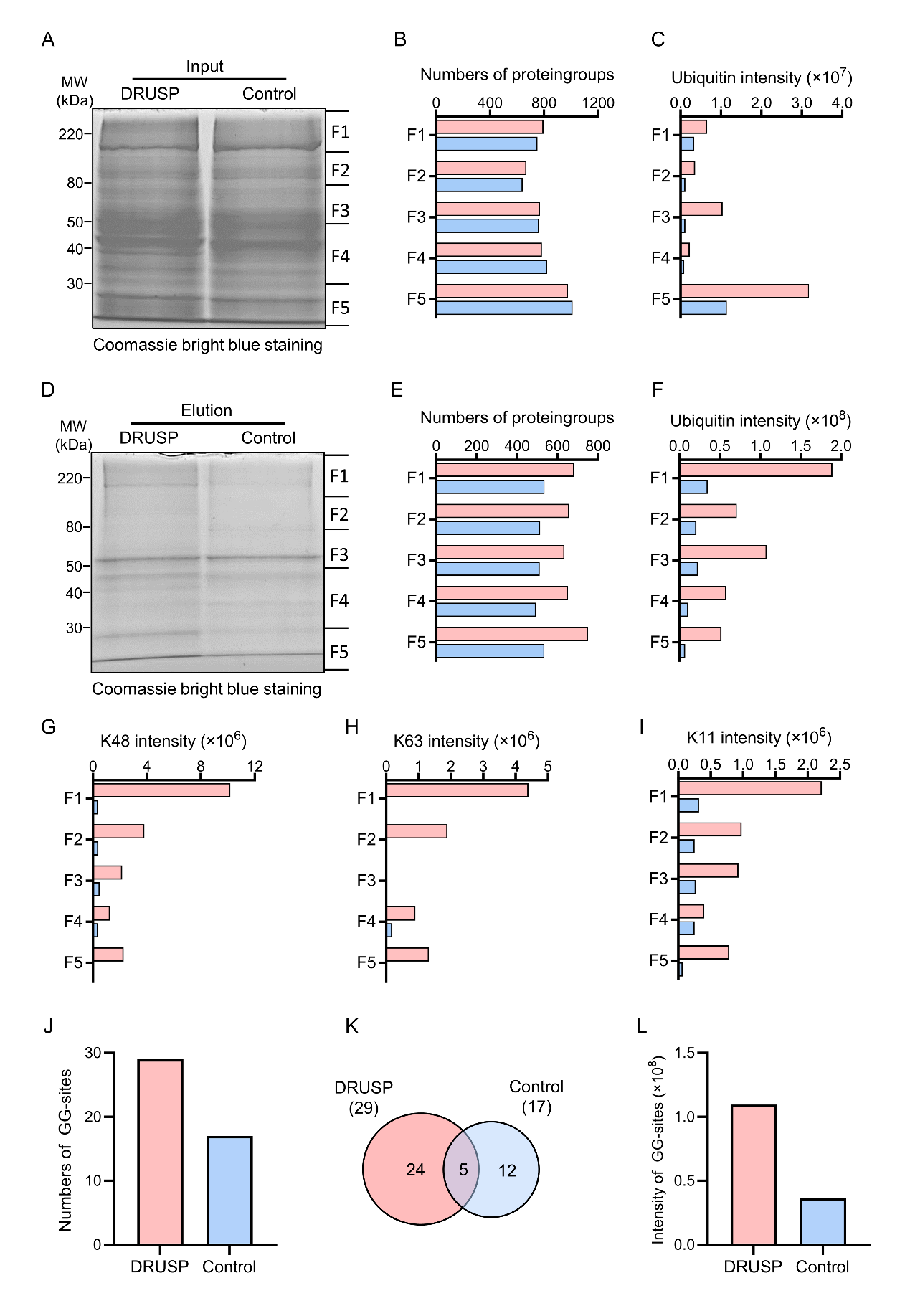


**Figure S5. The comparation of ubiquitination signal within input and elution samples derived from DRUSP and Control method.**

(A) The gel image and distribution area of each fraction in the Input samples of DRUSP and Control is showed.

(B) The number of protein groups identified in each fraction of panel A.

(C) The overall identification of ubiquitination signal in each component of panel A.

(D) The gel image and distribution area of each fraction in the Elution samples of DRUSP and Control was showed.

(E) The number of protein groups identified in each component of panel D.

(F) The overall identification of ubiquitination signal in each component of panel D.

(G) - (I) Quantification of ubiquitin chains by each component of panel D, in order of K48, K63 and K11.

(J) The number of K-ε-GG modified sites identified by DRSUP and Control method.

(K) Venn diagrams of the ubiquitination sites identified under both methods.

(L) The total signal strength of K-ε-GG modified sites identified by two methods.


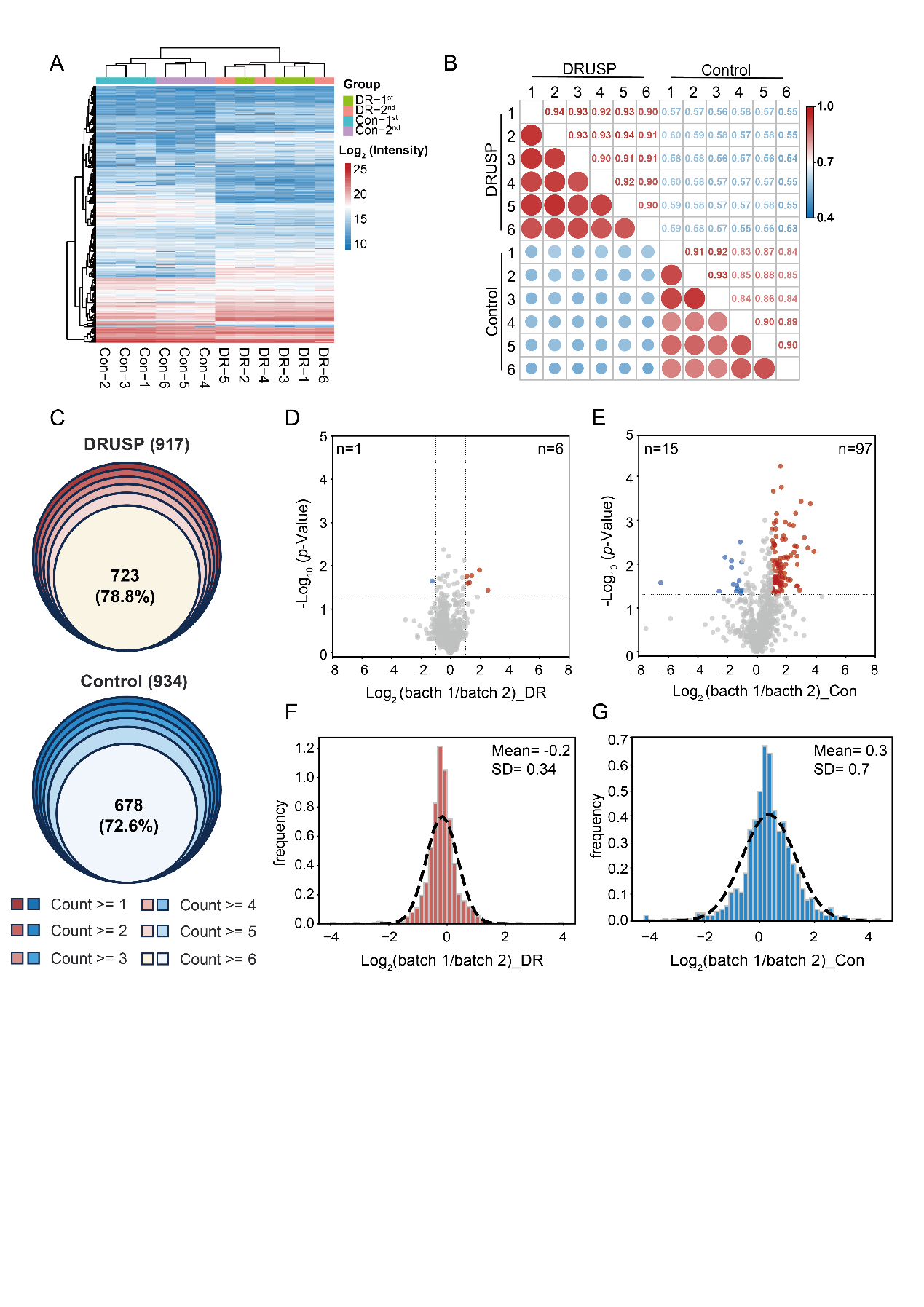


**Figure S6. DRUSP showed a higher quantitative accuracy and reproducibility compared to Control method**

(A) Hierarchical cluster analysis between batches of denatured DRUSP and Control conditions.

(B) Correlation analysis of log_2_ protein intensity between each group.

(C) The numbers of proteins with different identification times.

(D & E) Volcano maps of different proteins screened under DRUSP (D) and Control (E) groups.

(F & G) The distribution of log_2_ batch 1 to 2 ratios under DRUSP (F) and Control (G) groups.


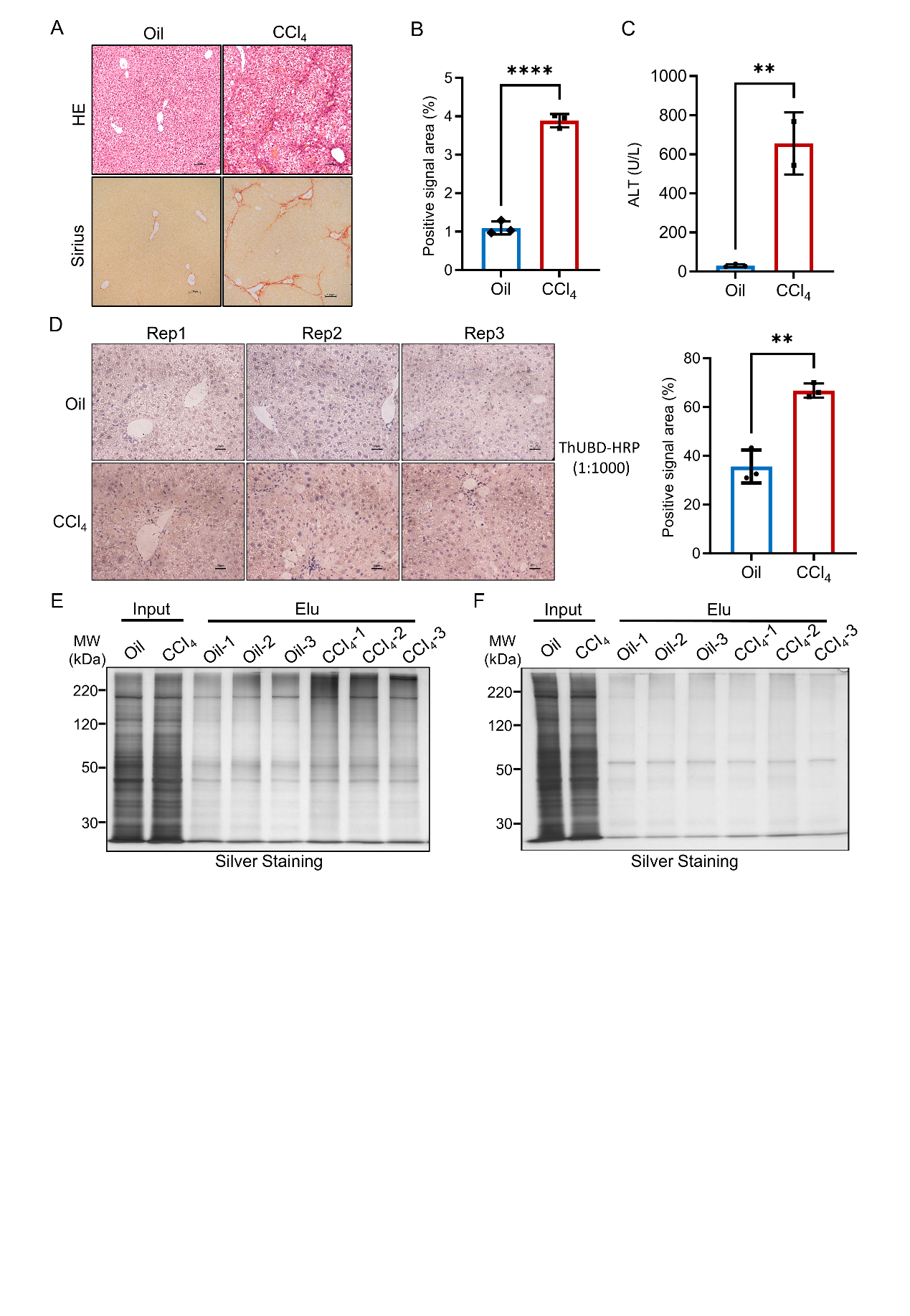


**Figure S7. Mouse liver fibrosis models were validated and used for ubiquitinome analysis**

(A) HE staining and Sirius red staining of mouse liver fibrosis before and after CCl_4_ treatment.

(B) A bar chart shows the positive area of Sirius red. The dots represent three biological repeats. **** *p*-value < 0.001.

(C) The average level of serum ALT measured by automatic biochemical analyzer (HITACHI 7180). ** *p*-value < 0.01

(D) IHC of *in situ* ubiquitination at tissue level was verified by ThUBD-HRP. The dilution ratio of ThUBD-HRP was 1: 1000. The histogram is quantitative information about the positive regions of IHC. ** *p*-value < 0.01.

(E & F) Silver staining was performed to verify the purification of ubiquitinated proteins in the early hepatic fibrosis model under DRUSP (E) and Control (F) conditions.


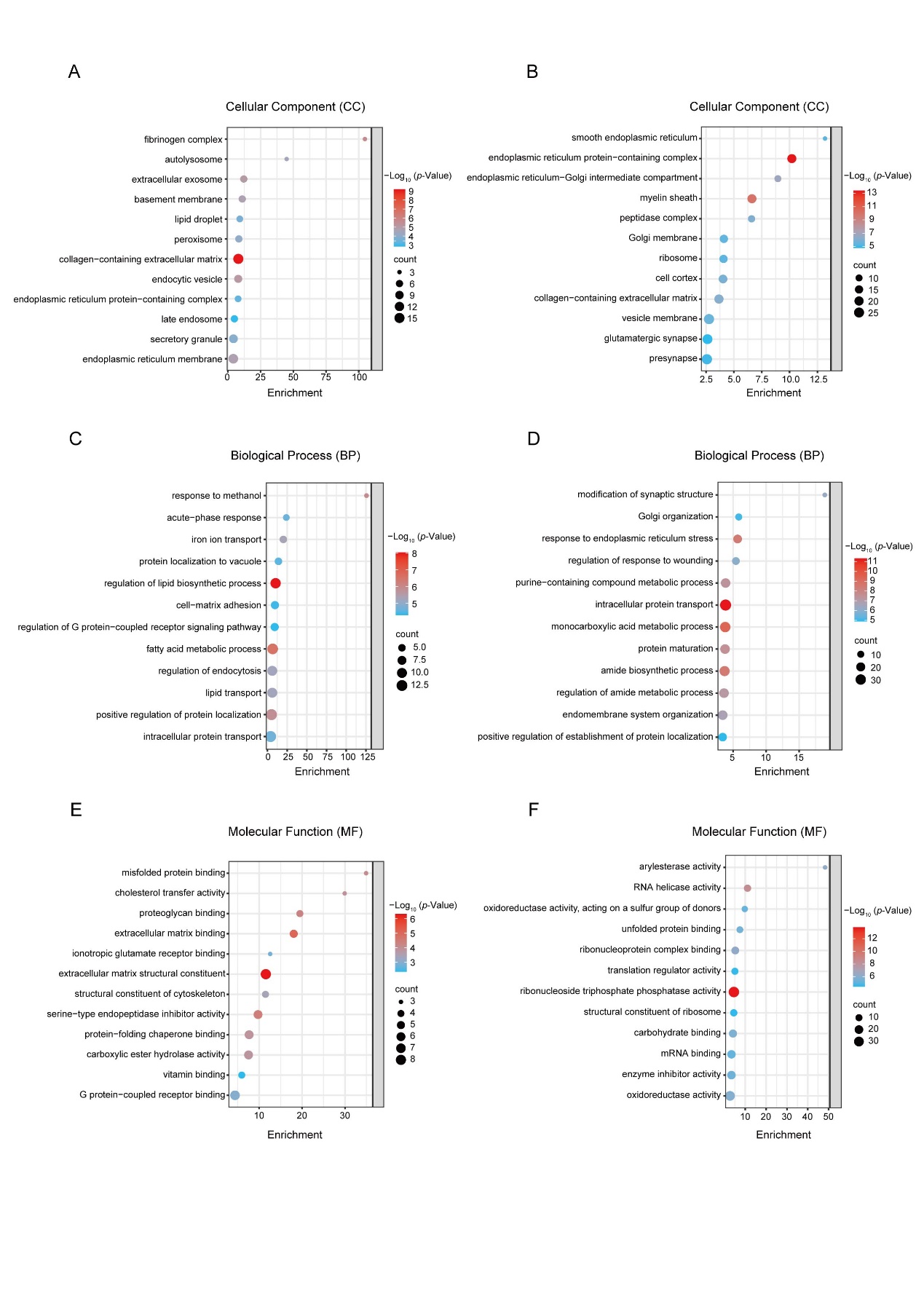


**Figure S8. GO enrichment of common and unique upregulated proteins from DRUSP and Control dataset in early-stage liver fibrosis**

(A & B). GO analysis of Cellular Component (CC) of common (A) and unique (B) upregulated proteins from Figure 7C. The color represented the *p*-Value, and the circle size represents the number of genes enriched.

(C & D). GO analysis of Biological Process (BP) of common (C) and unique (D) upregulated proteins from Figure 7C.

(E & F). GO analysis of Molecular Function (MF) of common (E) and unique (F) upregulated proteins from Figure 7C.
